# Gut microbiome diversity, genus-level variation, and associations with clinical features in critically ill patients

**DOI:** 10.3389/fmed.2026.1903736

**Published:** 2026-09-23

**Authors:** Yibing Wang, Peng Guo, Jie Shen

**Affiliations:** Center of Emergency and Critical Medicine, Jinshan Hospital of Fudan University, Shanghai, China

**Keywords:** critical illness, gut microbiome, inflammation, microbial diversity, sepsis

## Abstract

**Background:**

Gut microbiota alterations are common in critical illness, but differences between critically ill patients with and without sepsis and their clinical relevance remain incompletely understood. We investigated gut microbial diversity, genus-level differences and their associations with inflammatory profiles and clinical outcomes in critically ill patients.

**Methods:**

This single-centre case-control study enrolled adult critically ill patients grouped by Sepsis-3 criteria. Gut microbiota was profiled by 16S rRNA sequencing. The primary M2 model, adjusted for age, intra-abdominal infection, pre-sampling anti-anaerobic antibiotic exposure, APACHE II score, and chronic kidney disease, was used for all adjusted analyses. Alpha diversity was analyzed with HC3-robust linear regression; beta diversity with PERMANOVA, PERMDISP, and dbRDA. Genus-level primary discovery used Wilcoxon rank-sum with Benjamini-Hochberg FDR and |log2 fold change| threshold. Complementary sensitivity analyses used fully adjusted MaAsLin2 and ANCOM-BC2 with pseudo-count sensitivity. Associations of the four Wilcoxon-identified genera with clinical variables were assessed via partial Spearman correlations, logistic regression, and longitudinal mixed-effects models.

**Results:**

Of 103 participants, 68 had sepsis and 35 did not. After M2 adjustment, Shannon, Simpson, and Pielou evenness were lower in sepsis (*β* = −0.60, −0.54, −0.60; all *q* = 0.025), whereas Chao1 richness and observed species did not differ. Bray-Curtis and Jaccard group effects did not meet FDR threshold. Wilcoxon identified higher Lactococcus_A and lower Ezakiella, Fenollaria, Peptoniphilus_B in sepsis. ANCOM-BC2 signals were not robust to pseudo-count sensitivity, and MaAsLin2 identified no significant genus (min *q* = 0.333), indicating genus-level findings inconsistent across methods. No cross-sectional clinical association met significance after FDR correction. Longitudinal analyses found time-by-genus interactions: Fenollaria with CRP (*q* = 0.010), Peptoniphilus_B with PCT (*q* = 0.042) and CRP (*q* = 0.010).

**Conclusions:**

Sepsis was associated with lower values for selected alpha-diversity measures, whereas differences in overall community composition were modest. Four genera met the primary FDR- and effect-size-adjusted Wilcoxon criteria, but genus-level differences were not consistently supported across Wilcoxon, ANCOM-BC2 and MaAsLin2, indicating these are exploratory signals rather than confirmed candidates. Associations with clinical features and inflammatory markers were limited; therefore, these findings should be considered exploratory and validated using multiple analytical approaches in independent cohorts.

## 1 Introduction

Critically ill patients, including those with sepsis, commonly experience immune dysregulation, multiple-organ dysfunction and a high risk of adverse outcomes (1). In recent years, the intestine has been recognized not only as an organ responsible for nutrient absorption but also as an important site for host immune regulation, maintenance of barrier function, and microbial homeostasis (1). Interactions between the gut microbiota and host inflammatory responses may therefore influence the progression of critical illness.

Previous studies have described reduced alpha diversity, depletion of beneficial commensal taxa and enrichment of potentially pathogenic bacteria in sepsis (2). In critically ill patients, anti-anaerobic antibiotic exposure has also been associated with gut microbial disruption and expansion of Enterobacteriaceae (3). Antibiotic exposure, shock-related intestinal hypoperfusion, nutritional changes, mechanical ventilation, and organ support therapies may all contribute to intestinal microecological disruption in critically ill patients (1, 4). Gut microbiota dysbiosis may be associated with impaired intestinal barrier integrity, translocation of bacteria, and amplified systemic inflammation and organ dysfunction through a gut microbiota-intestinal barrier-systemic inflammation axis (5). Therefore, alterations in the gut microbiota are increasingly regarded as an important component of the pathophysiological process in critically ill patients.

However, the key question is no longer whether gut microbiota dysbiosis occurs in critically ill patients, but which microbial alterations are clinically relevant. Although reduced microbial richness and diversity have been associated with adverse outcomes in critical illness, it remains unclear whether specific genus-level changes correspond to different inflammatory states. In particular, the relationships between altered gut genera, systemic inflammatory markers, and circulating indicators of intestinal injury have not been fully characterized in critically ill patients with sepsis.

Based on these considerations, this single-centre case-control study enrolled critically ill patients with and without sepsis to characterize gut microbial diversity and genus-level differences within the broader context of critical illness. We further examined whether the identified genus-level differences were associated with systemic inflammatory responses, circulating intestinal-barrier-related biomarkers, longitudinal inflammatory profiles and short-term clinical outcomes. We used Wilcoxon rank-sum testing as the primary two-group discovery analysis and applied MaAsLin2 and ANCOM-BC2 as complementary sensitivity analyses to assess covariate-adjusted and compositional robustness.

## 2 Materials and methods

### 2.1 Study design and ethical approval

This single-centre case-control study was conducted in the Department of Critical Care Medicine, Jinshan Hospital, Fudan University, between January 2024 and January 2025. The protocol was approved by the Ethics Committee of Jinshan Hospital, Fudan University. Written informed consent was obtained from each participant or their legally authorised representative before enrolment, in accordance with the Declaration of Helsinki.

### 2.2 Study participants and grouping

Adults admitted to the intensive care unit (ICU) of Jinshan Hospital during the study period were screened for eligibility. Patients were grouped according to the presence or absence of sepsis based on Sepsis-3 criteria. Sepsis was defined as suspected or confirmed infection with an acute increase in Sequential Organ Failure Assessment (SOFA) score of ≥2 points. Patients who did not meet these criteria but were critically ill were assigned to the non-sepsis (critically ill control) group.

### 2.3 Inclusion and exclusion criteria

Inclusion criteria: (1) Age > 18 years; (2) For sepsis group: meeting the Sepsis-3 diagnostic criteria; (3) For non-sepsis group: ICU inpatients (not meeting sepsis criteria) during the same period. (4) Provision of a qualified anal swab at enrolment.

Exclusion criteria: (1) Life expectancy ≤ 48 hours; (2) Pregnant or lactating women; (3) Active autoimmune disease or history of autoimmune disease; (4) Human immunodeficiency virus infection or other immunosuppressive conditions; (5) Participation in another drug clinical trial within the previous month; (6) Inability to obtain a qualified anal swab sample.

### 2.4 Data collection and clinical variables

At enrolment (T0), demographic data, comorbidities, infection source, initial antibiotic coverage, documented antimicrobial exposure before microbiota sampling, nutritional route at the time of microbiota sampling, and disease severity scores [Acute Physiology and Chronic Health Evaluation II (APACHE II) and SOFA] were recorded. Conventional inflammatory markers [C-reactive protein (CRP), procalcitonin (PCT), serum amyloid A (SAA), and white blood cell count (WBC)] were measured at T0, days 1–3 (T1) and days 3–7 (T2) after enrolment. Baseline inflammatory cytokines (IL-2, IL-4, IL-6, IL-10, TNF-α) and circulating intestinal-barrier-related biomarkers [diamine oxidase (DAO), lipopolysaccharide-binding protein (LBP), and zonulin] were measured at T0. Gut microbiota features were assessed using 16S rRNA sequencing of anal swab samples. Clinical complications and outcome-related variables included septic shock, acute respiratory distress syndrome (ARDS), acute kidney injury (AKI), acute gastrointestinal injury (AGI), 28-day all-cause mortality, in-hospital mortality, ICU and hospital lengths of stay, and durations of mechanical ventilation and vasoactive medication use.

#### 2.4.1 Definitions of clinical complications and outcomes

Clinical complications were assessed from enrolment through the index ICU admission. Septic shock was defined according to the Sepsis-3 criteria as sepsis requiring vasopressor therapy to maintain a mean arterial pressure of ≥65 mmHg together with a serum lactate concentration >2 mmol/L despite adequate volume resuscitation. Acute respiratory distress syndrome (ARDS) was diagnosed according to the Berlin definition. Acute kidney injury (AKI) was defined according to the Kidney Disease: Improving Global Outcomes criteria as an increase in serum creatinine of ≥0.3 mg/dL within 48 h, an increase to ≥1.5 times the baseline value within 7 days, or urine output <0.5 mL/kg/h for at least 6 h.

Acute gastrointestinal injury (AGI) was classified according to the European Society of Intensive Care Medicine grading system. For the binary analysis, the occurrence of any AGI grade from I to IV during the index ICU admission was coded as present. Maximum AGI grade and AGI duration were not analysed as separate outcomes in the primary clinical-association analyses.

Twenty-eight-day all-cause mortality was defined as death from any cause within 28 days after enrolment. ICU and hospital lengths of stay were calculated from the corresponding admission dates until discharge or death. Durations of mechanical ventilation and vasoactive medication use were defined as the cumulative durations of the corresponding treatments during the index hospitalisation.

### 2.5 Sample collection and laboratory measurements

#### 2.5.1 Anal swab sampling and 16S rRNA sequencing

All microbiota specimens included in the present cohort were collected using anal swabs at enrolment. Anal swab samples were collected at enrolment using a standardized procedure that was applied consistently to all participants. Sterile cotton swabs moistened with normal saline were used for sample collection. The samples were transported on dry ice to Sinobestbio (Shanghai Youxuan Biotechnology Co., Ltd., Shanghai, China; http://www.sinobestbio.com). The company performed DNA extraction, PCR amplification, library preparation, sequencing and the initial bioinformatic processing. The procedures are described below.

Microbial DNA was extracted directly from the anal swabs using the company's standard protocol for faecal samples and the MagBeads FastDNA Kit for Soil (catalogue no. 116564384; MP Biomedicals, CA, USA). DNA concentration and integrity were evaluated using a NanoDrop spectrophotometer and 0.8% agarose gel electrophoresis.

The V3–V4 region of the bacterial 16S rRNA gene was amplified with the primers 338F (5′-ACTCCTACGGGAGGCAGCA-3′) and 806R (5′-GGACTACHVGGGTWTCTAAT-3′). PCR was performed using Q5 High-Fidelity DNA Polymerase (NEB, M0491L). The amplification programme consisted of 5 min at 98°C, followed by 25 cycles of 30 s at 98°C, 30 s at 52°C and 45 s at 72°C, with a final extension for 5 min at 72°C.

The PCR products were separated on a 2% agarose gel and purified using an Axygen gel extraction kit. Purified amplicons were quantified with the Quant-iT PicoGreen dsDNA Assay Kit and then pooled. Libraries were prepared using the TruSeq Nano DNA LT Library Prep Kit. Library concentration and fragment distribution were assessed using a Qubit 4 fluorometer and an Agilent 2100 Bioanalyzer. Sequencing was performed on an Illumina NovaSeq 6000 using the NovaSeq 6000 SP Reagent Kit with 500 cycles, producing 2 × 250 bp paired-end reads.

#### 2.5.2 Bioinformatic processing and sequencing-depth assessment

Sinobestbio also performed the initial bioinformatic analysis. Raw sequencing data were demultiplexed and processed using QIIME 2 version 2024.5. Primer sequences were removed with the q2-cutadapt trim-paired workflow.

The remaining reads were processed with the q2-dada2 denoise-paired workflow, including quality filtering, denoising, paired-read merging, chimera removal and inference of amplicon sequence variants (ASVs). The resulting representative sequences and feature table were subsequently imported into QIIME 2 version 2022.11.1 for feature filtering and taxonomic classification. ASV counts were aggregated at the genus level. The Wilcoxon analyses used relative abundances derived from the unrarefied genus-level count matrix, whereas ANCOM-BC2 used the corresponding unrarefied integer counts. MaAsLin2 used the same genus-level count matrix with total-sum scaling and log transformation. Cumulative-sum scaling and centred log-ratio transformation were not applied in the Wilcoxon or ANCOM-BC2 analyses.

Taxonomy was assigned using q2-feature-classifier classify-sklearn in QIIME 2 version 2022.11.1. The Naive Bayes classifier was trained using the Greengenes2 database, release 2022.10. Reference sequences were restricted to the region amplified by the 338F and 806R primers, and the classification confidence threshold was 0.7.

Before rarefaction, sequencing depths ranged from 37,261 to 108,145 reads per sample in the Non-sepsis group and from 38,216 to 98,644 reads per sample in the Sepsis group. Alpha-diversity indices were obtained using QIIME 2 alpha-rarefaction from the unrarefied ASV table. Ten evenly spaced sequencing depths between 10 and 35,397 reads were evaluated with 10 iterations at each depth, and the mean value at the maximum depth of 35,397 reads was used for analysis. Beta-diversity distance matrices were generated using the QIIME 2 core-metrics-phylogenetic workflow after rarefaction without replacement to 35,397 reads per sample.

Zero values were retained when relative abundances were calculated. When log_2_ fold changes were estimated, a pseudocount of 1 × 10^−5^ was added to the mean relative abundance of each group.

#### 2.5.3 Blood sampling and biomarker measurement

Serum IL-2, IL-4, IL-6, IL-10, TNF-α, DAO, LBP and zonulin concentrations were measured using commercial human ELISA kits (KeyGEN BioTECH, Nanjing, China) according to the manufacturer's instructions. Standard curves were generated using the manufacturer-supplied calibrators, and concentrations were calculated from blank-corrected optical-density values, with dilution correction applied where required. CRP, PCT, SAA and WBC were measured at T0, T1 and T2 using routine clinical laboratory methods.

### 2.6 Gut microbiota analysis

#### 2.6.1 Alpha- and beta-diversity analyses

Alpha diversity was quantified using the Shannon, Chao1, observed-species, Simpson and Pielou evenness indices. Shannon was analysed without transformation; Chao1 and observed species were log-transformed; and Simpson and Pielou evenness were logit-transformed. Each metric was standardized before linear regression. HC3 robust standard errors were used to calculate 95% confidence intervals and *P* values. Benjamini-Hochberg correction was applied across the five metrics within each model.

Beta diversity was assessed using Bray-Curtis, Jaccard, weighted UniFrac and unweighted UniFrac distances. Lingoes-corrected principal coordinate analysis was used for visualization, with 80% normal data ellipses. Marginal PERMANOVA with 9,999 permutations estimated the Group partial *R*^2^ after model-specific adjustment using the M2 covariate set (age, intra-abdominal infection, documented pre-sampling anti-anaerobic antibiotic exposure, APACHE II score, and chronic kidney disease). PERMDISP tested homogeneity of group dispersion using bias-adjusted distances to centroids and 9,999 permutations. Benjamini-Hochberg correction was applied across the four distances within each model. Distance-based redundancy analysis (dbRDA) using the M2 covariate set was used to partition the variation in community composition, and components were tested using 9,999 permutations, with correction across the two Group-specific tests.

#### 2.6.2 Genus-level differential-abundance analyses

Genus-level differential abundance was evaluated using three approaches: Wilcoxon rank-sum testing, ANCOM-BC2 and MaAsLin2. Analyses were restricted to genera with a prevalence of at least 10% and a cohort-wide mean relative abundance of at least 0.01% (*n* = 190).

Two-sided Wilcoxon rank-sum tests were used to compare unrarefied genus-level relative abundances between the Sepsis and Non-sepsis groups, with Benjamini-Hochberg correction applied across the 190 tested genera. Candidates were defined by *q* < 0.05 and |log_2_ fold change| > 1. A pseudocount of 1 × 10^−5^ was added only to group means when calculating log_2_ fold changes.

ANCOM-BC2 used the corresponding unrarefied integer counts and model-specific covariates. Continuous covariates were standardized, structural-zero detection and pseudo-count sensitivity were enabled, and Group coefficients were Benjamini-Hochberg corrected.

MaAsLin2 analysed the same counts using total-sum scaling, log transformation and linear models. Group and model-specific covariates were fixed effects, continuous covariates were standardized, and no random effects were included. Non-sepsis was the reference category and the minimum prevalence was 10%. Benjamini-Hochberg correction was applied within each model. Statistical significance was defined as *q* < 0.05; *q* < 0.25 was used only for supplementary visualization.

### 2.7 Clinical-association and longitudinal analyses

Wilcoxon-identified genera meeting the Wilcoxon criteria were examined in cross-sectional and longitudinal analyses. Continuous clinical features were analysed using partial Spearman correlations after rank transformation and covariate residualization. Binary complications and mortality were analysed using logistic regression, with odds ratios expressed per 1-standard-deviation increase in genus abundance rank. Septic shock was analysed only among patients with sepsis. Benjamini-Hochberg correction was applied separately within each clinical domain and model.

Longitudinal associations with PCT, CRP and WBC were evaluated using linear mixed-effects models. In these models, genus abundance was entered as a continuous, log1p-transformed, and standardized fixed effect. Baseline genus abundance was log1p-transformed and standardized. PCT and CRP were log1p-transformed, whereas WBC was untransformed; all markers were standardized using T0 parameters. Models included categorical time, genus abundance, their interaction, M2 covariates and a participant-specific random intercept. Participants required T0 and at least one follow-up measurement. Time-by-Genus interactions were tested by two-degree-of-freedom likelihood-ratio tests using maximum likelihood, with correction across 12 genus-marker combinations. Restricted maximum likelihood models estimated P75-versus-P25 contrasts.

### 2.8 Sample size estimation

The sample-size calculation was designed to support two principal analytical objectives: (1) comparison of gut microbial characteristics between critically ill patients with and without sepsis and (2) evaluation of associations between differentially abundant genera and clinical features. Based on the anticipated distribution of eligible patients in the ICU, a 2:1 allocation ratio between the Sepsis and Non-sepsis groups was assumed.

Using Fisher’s z-transformation, the minimum number of participants required to detect a moderate correlation coefficient (*r* = 0.30) with 80% power and a two-sided α of 0.05 was estimated as:

$$n={\left[\frac{Z_{\alpha /2}+Z_{\beta }}{0.5\times \mathrm{In}\left(\frac{1+r}{1-r}\right)}\right]}^{2}+3$$

For the between-group comparison, a two-sided test with an α level of 0.05, 80% power and a minimum standardized effect size of Cohen's *d* = 0.60 required 102 evaluable participants. For the genus-clinical feature association analysis, 84 participants were required to detect a Spearman correlation coefficient of *ρ* = 0.30 with 80% power and a two-sided α level of 0.05. The larger estimate was therefore used, resulting in a minimum required sample size of 102 evaluable participants.

### 2.9 Statistical analysis

Statistical analyses were performed using R software (version 4.5.2). Continuous variables were summarized as mean ± standard deviation or median with interquartile range, according to their distributional characteristics. Normally distributed continuous variables were compared between groups using Welch's *t*-test, whereas non-normally distributed variables were compared using the Mann–Whitney U test. Categorical variables were summarized as counts and percentages and compared using Pearson's *χ*^2^ test. Yates’ continuity correction was applied to 2 × 2 tables when all expected cell counts were at least 5; Fisher's exact test was used when any expected cell count was below 5.

Longitudinal changes in CRP, PCT, SAA and WBC were evaluated using linear mixed-effects models with a participant-specific random intercept. Group, categorical time and their interaction were included as fixed effects. CRP, PCT and SAA were log1p-transformed before modelling, whereas WBC was analysed without transformation. Between-group contrasts at individual time points were estimated from the fitted models. Benjamini-Hochberg correction was applied separately to the time-specific contrasts, overall group-by-time interaction tests, baseline cytokine comparisons and intestinal-barrier biomarker comparisons.

Genus-level differential abundance was evaluated using three approaches. The two-sided Wilcoxon rank-sum test was prespecified as the primary discovery analysis, with Benjamini-Hochberg (BH) false-discovery rate (FDR) correction (*q* < 0.05) and a |log2 fold change| > 1 applied jointly. To assess the robustness of the primary findings, two complementary sensitivity frameworks were used: fully adjusted MaAsLin2, and ANCOM-BC2 with pseudo-count sensitivity testing. Multivariable adjustment (M2) was applied consistently to all relevant adjusted models, including alpha-diversity regression, PERMANOVA, dbRDA, ANCOM-BC2, MaAsLin2, clinical-association, and longitudinal mixed-effects analyses. The M2 covariate set comprised age, intra-abdominal infection, documented pre-sampling anti-anaerobic antibiotic exposure, APACHE II score, and chronic kidney disease.

M0 was unadjusted, whereas M1 adjusted for age, sex and body mass index. S1 replaced APACHE II with the baseline SOFA score. S2 excluded participants with CKD and adjusted for age, intra-abdominal infection, pre-sampling anti-anaerobic exposure and APACHE II score. R1 was restricted to participants without intra-abdominal infection and adjusted for age, pre-sampling anti-anaerobic exposure, APACHE II score and CKD. M0, M1, S1, S2 and R1 served as sensitivity analyses. An additional longitudinal sensitivity model added Group and Group-by-Time terms to M2.

Effect estimates are reported with 95% confidence intervals. Depending on the analysis, effect measures comprised standardized regression coefficients, PERMANOVA partial *R*^2^ values, dbRDA adjusted *R*^2^ values, partial Spearman *ρ* coefficients, odds ratios and Time-by-Genus interaction statistics. Alpha-diversity regression used HC3 heteroscedasticity-consistent standard errors. Microbial community analyses were performed using vegan; differential-abundance analyses used ANCOM-BC2 and MaAsLin2; and longitudinal models and estimated contrasts used lme4, lmerTest and emmeans.

All statistical tests were two-sided. Benjamini-Hochberg false-discovery rate correction was performed within each prespecified analysis family, including the five alpha-diversity metrics; four beta-diversity distance measures; two dbRDA Group-specific tests; genera tested within each differential-abundance method; associations within each clinical domain and model; and the 12 longitudinal Time-by-Genus interaction tests within each analysis set. Statistical significance was defined as an FDR-adjusted *q* value < 0.05.

## 3 Results

### 3.1 Clinical characteristics of the study population

A total of 103 critically ill patients were enrolled, including 68 patients with sepsis and 35 non-septic controls. All microbiota specimens were obtained using anal swabs in both the sepsis group (68/68, 100%) and the non-sepsis group (35/35, 100%). As shown in Table 1, no significant between-group differences were observed in age, sex, BMI, hypertension, diabetes mellitus, chronic obstructive pulmonary disease, coronary heart disease, liver cirrhosis, or cerebrovascular disease. A higher proportion of patients in the non-sepsis group had chronic kidney disease than in the sepsis group (11.4% vs. 1.5%, *P* = 0.044). Patients with sepsis had higher APACHE II scores than the non-sepsis group (21.94 ± 8.35 vs. 16.74 ± 6.59, *P* < 0.001). Median SOFA scores were also higher in the sepsis group [9.00 (7.00, 12.00) vs. 4.00 (2.00, 5.00), *P* < 0.001]. The overall distribution of primary infection sites did not differ significantly between the groups (*P* = 0.059). Pulmonary infections accounted for 38.2% and 62.9% of cases in the sepsis and non-sepsis groups, respectively, whereas the corresponding proportions of intra-abdominal infections were 44.1% and 20.0%. Acute kidney injury was more frequent in the sepsis group (55.9% vs. 22.9%, *P* = 0.003). The proportions of acute respiratory distress syndrome did not differ significantly between the groups, nor did the proportion of acute gastrointestinal injury (69.1% vs. 48.6%, *P* = 0.068). Septic shock was recorded in 33 patients (48.5%) in the sepsis group. Nutritional route at the time of microbiota sampling was documented for all participants. No patient in either group was receiving parenteral nutrition at the time of microbiota sampling.

**Table 1:** Baseline demographic and clinical characteristics of critically ill patients with and without sepsis.

Characteristics	Overall (*n* = 103)	Sepsis (*n* = 68)	Non-sepsis (*n* = 35)	Statistic	*P*
Age (years)	78.00 (69.00, 84.00)	76.50 (69.00, 83.25)	78.00 (65.00, 86.00)	*U* = 1145	0.757
Male sex [*n* (Male, %)]	71 (68.9%)	47 (69.1%)	24 (68.6%)	*χ^2^* = 0.000	1.000
BMI (kg/m^2^)	22.86 (20.26, 24.87)	22.14 (19.86, 24.54)	23.66 (22.00, 25.08)	*U* = 950	0.096
Hypertension (*n*, %)	71 (68.9%)	48 (70.6%)	23 (65.7%)	*χ^2^* = 0.079	0.778
Diabetes mellitus (*n*, %)	40 (38.8%)	25 (36.8%)	15 (42.9%)	*χ^2^* = 0.150	0.698
Chronic kidney disease (*n*, %)	5 (4.9%)	1 (1.5%)	4 (11.4%)	–	0.044*
Chronic obstructive pulmonary disease (*n*, %)	16 (15.5%)	9 (13.2%)	7 (20.0%)	*χ^2^* = 0.373	0.541
Coronary heart disease (*n*, %)	25 (24.3%)	15 (22.1%)	10 (28.6%)	*χ^2^* = 0.238	0.626
Liver cirrhosis (*n*, %)	2 (1.9%)	2 (2.9%)	0 (0.0%)	–	0.547
Cerebrovascular disease (*n*, %)	33 (32.0%)	21 (30.9%)	12 (34.3%)	*χ^2^* = 0.016	0.898
APACHE II score	20.17 ± 8.15	21.94 ± 8.35	16.74 ± 6.59	*t* = 3.453	<0.001***
SOFA score at enrolment	7.00 (5.00, 11.00)	9.00 (7.00, 12.00)	4.00 (2.00, 5.00)	*U* = 2160	<0.001***
Primary infection site				–	0.059
Pulmonary (*n*, %)	48 (46.6%)	26 (38.2%)	22 (62.9%)		
Intra-abdominal (*n*, %)	37 (35.9%)	30 (44.1%)	7 (20.0%)		
Urinary tract (*n*, %)	8 (7.8%)	6 (8.8%)	2 (5.7%)		
Other (*n*, %)	10 (9.7%)	6 (8.8%)	4 (11.4%)		
Septic shock (*n*, %)	33 (32.0%)	33 (48.5%)	0 (0.0%)	*χ^2^* = 22.814	<0.001***
Acute respiratory distress syndrome (*n*, %)	44 (42.7%)	31 (45.6%)	13 (37.1%)	*χ^2^* = 0.373	0.542
Acute kidney injury (*n*, %)	46 (44.7%)	38 (55.9%)	8 (22.9%)	*χ^2^* = 8.905	0.003**
Acute gastrointestinal injury (*n*, %)	64 (62.1%)	47 (69.1%)	17 (48.6%)	*χ^2^* = 3.319	0.068

Continuous variables are presented as mean ± standard deviation or median (P25, P75), as appropriate; categorical variables are presented as *n* (%).

Between-group comparisons were performed using Welch's *t*-test, the Mann–Whitney U test, Pearson's chi-square test with Yates’ continuity correction, or Fisher's exact test, as appropriate.

Other infection sites included skin and soft-tissue infections, central nervous system infections, bloodstream infections, and infections with an unidentified primary site.

BMI, body mass index; APACHE II, Acute Physiology and Chronic Health Evaluation II; SOFA, Sequential Organ Failure Assessment; ARDS, acute respiratory distress syndrome.

* *P* < 0.05.

** *P* < 0.01.

*** *P* < 0.001.

As shown in Table 2, antibiotic use on ICU day 1 and before microbiota sampling was more frequent in the sepsis group (97.1% vs. 85.7%, *P* = 0.043), whereas the interval from antibiotic initiation to sampling did not differ between the groups (*P* = 0.446). Anti-anaerobic coverage and carbapenem exposure were more frequent, while cephalosporin exposure was less frequent, in the sepsis group (all *P* < 0.001). Among patients receiving antibiotics before sampling, the initial regimen remained unchanged less frequently (56.1% vs. 86.7%, *P* = 0.007), and combination therapy was more frequent (19.1% vs. 0%, *P* = 0.004) in the sepsis group. Vasoactive drug use was also more frequent in the sepsis group (52.9% vs. 25.7%, *P* = 0.015), whereas antibiotic duration, mechanical ventilation, and ICU and hospital lengths of stay did not differ. The 28-day mortality rates were 50.0% and 28.6%, respectively (*P* = 0.061).

**Table 2:** Clinical management and outcomes of critically ill patients with and without sepsis.

Characteristics	Overall	Sepsis	Non-sepsis	Statistic	*P*
Antibiotic exposure on ICU day 1 and before microbiota sampling, *n* (%)	96 (93.2%)	66 (97.1%)	30 (85.7%)	–	0.043*
Interval from antibiotic initiation to microbiota sampling, h	4.00 (2.00, 7.00)	4.00 (2.00, 8.50)	3.50 (2.00, 5.00)	*U* *=* 1086	0.446
Anti-anaerobic antibiotic coverage before sampling, *n* (%)	72 (69.9%)	60 (88.2%)	12 (34.3%)	*χ^2^* *=* 29.454	<0.001***
Carbapenem exposure before sampling, *n* (%)	66 (64.1%)	57 (83.8%)	9 (25.7%)	*χ^2^* *=* 31.419	<0.001***
Cephalosporin exposure before sampling, *n* (%)	21 (20.4%)	4 (5.9%)	17 (48.6%)	*χ^2^* *=* 23.379	<0.001***
β-lactam/β-lactamase inhibitor exposure before sampling, *n* (%)	5 (4.9%)	2 (2.9%)	3 (8.6%)	–	0.334
Exposure to other antibiotic classes before sampling, *n* (%)	8 (7.8%)	7 (10.3%)	1 (2.9%)	–	0.260
Initial empirical antibiotic regimen unchanged within 7 days, *n*/*N* (%)	63/96 (65.6%)	37/66 (56.1%)	26/30 (86.7%)	*χ^2^* *=* 7.261	0.007**
Combination antibiotic therapy within 7 days, *n* (%)	13 (12.6%)	13 (19.1%)	0 (0.0%)	–	0.004**
Total duration of antibiotic use, days	8.00 (5.00, 14.00)	8.50 (5.75, 14.25)	7.00 (5.00, 10.00)	*U* *=* 1333	0.319
Mechanical ventilation, *n* (%)	70 (68.0%)	45 (66.2%)	25 (71.4%)	*χ^2^* *=* 0.101	0.750
Duration of mechanical ventilation, days	5.50 (3.00, 14.00)	6.00 (3.00, 15.00)	5.00 (3.00, 7.00)	*U* *=* 610	0.558
Vasoactive drug use, *n* (%)	45 (43.7%)	36 (52.9%)	9 (25.7%)	*χ^2^* *=* 5.900	0.015*
Duration of vasoactive drug use, days	4.00 (3.00, 4.00)	4.00 (3.00, 4.00)	4.00 (3.00, 4.00)	*U* *=* 160	0.965
28-day mortality, *n* (%)	44 (42.7%)	34 (50.0%)	10 (28.6%)	*χ^2^* *=* 3.505	0.061
ICU length of stay, days	7.00 (5.00, 15.00)	7.00 (5.00, 14.00)	7.00 (5.50, 17.00)	*U* *=* 1210	0.894
Hospital length of stay, days	18.00 (10.00, 25.50)	16.00 (8.00, 24.00)	20.00 (11.50, 28.50)	*U* *=* 1026	0.253

Data are presented as *n* (%) or median (P25, P75), unless otherwise indicated.

The recorded antibiotic exposure status on ICU day 1 was identical to that before microbiota sampling in this cohort; therefore, the two variables are presented in a single row.

The interval from antibiotic initiation to microbiota sampling was calculated only among patients who received antibiotics before sampling (overall, *n* = 96; sepsis, *n* = 66; non-sepsis, *n* = 30).

Exposure to other antibiotic classes included tigecyclines, fluoroquinolones, polymyxins, glycopeptides and oxacephems. Antibiotic-class categories were not mutually exclusive because patients receiving combination therapy could be included in more than one category.

An unchanged initial empirical antibiotic regimen was defined as no change in the constituent antibacterial agents during the first 7 days.

Combination antibiotic therapy was defined as the concurrent administration of at least two systemic antibacterial agents during the first 7 days.

Mechanical ventilation duration was calculated only among mechanically ventilated patients (overall, *n* = 70; sepsis, *n* = 45; non-sepsis, *n* = 25). Vasoactive drug duration was calculated only among patients receiving vasoactive drugs (overall, *n* = 45; sepsis, *n* = 36; non-sepsis, *n* = 9).

Between-group comparisons were performed using the Mann–Whitney U test, Pearson's chi-square test with Yates’ continuity correction, or Fisher's exact test, as appropriate. For Fisher's exact test, no test statistic is reported and the Statistic column is shown as an em dash. All *P* values are two-sided.

ICU, intensive care unit; P25, 25th percentile; P75, 75th percentile.

* *P* < 0.05.

** *P* < 0.01.

*** *P* < 0.001.

As shown in Table 3, CRP, PCT and SAA levels at enrolment were higher in patients with sepsis than in non-septic patients after Benjamini-Hochberg correction. PCT remained higher in the sepsis group at T1, whereas the between-group differences in CRP at T1 and T2, PCT at T2, and SAA at T1 and T2 were not statistically significant after correction. Significant group-by-time interactions were observed for CRP, PCT and SAA, but not for WBC count. At enrolment, IL-6, IL-10 and TNF-α levels were higher in patients with sepsis (BH-adjusted *P* < 0.001, *P* = 0.009 and *P* = 0.006, respectively), whereas IL-2 and IL-4 did not differ significantly between the groups. No significant between-group differences were observed in DAO, LBP or zonulin.

**Table 3:** Inflammatory markers and circulating intestinal-barrier-related biomarkers in patients with and without sepsis.

Characteristics	Overall	Sepsis	Non-sepsis	Statistic	*P* value	BH-adjusted *P* value
Conventional inflammatory markers						
C-reactive protein (mg/L)						
T0	87.25 (27.62, 178.00); *n* = 103	115.23 (70.47, 209.16); *n* = 68	22.74 (5.05, 47.12); *n* = 35	*U* = 2047	<0.001	<0.001***
T1	68.87 (28.63, 130.65); *n* = 101	74.96 (39.31, 166.74); *n* = 67	53.88 (23.68, 88.78); *n* = 34	*U* = 1448	0.027	0.053
T2	48.97 (17.80, 80.63); *n* = 83	56.07 (35.54, 83.09); *n* = 54	27.10 (14.55, 67.14); *n* = 29	*U* = 980	0.061	0.104
Group-by-time interaction				*F* = 22.461	<0.001	<0.001***
Procalcitonin (ng/mL)						
T0	2.46 (0.36, 18.73); *n* = 103	6.99 (1.33, 28.62); *n* = 68	0.35 (0.13, 1.01); *n* = 35	*U* = 1937	<0.001	<0.001***
T1	1.60 (0.37, 10.10); *n* = 101	3.76 (0.72, 13.87); *n* = 67	0.49 (0.17, 1.75); *n* = 34	*U* = 1655	<0.001	<0.001***
T2	0.69 (0.19, 2.37); *n* = 82	0.79 (0.35, 2.86); *n* = 54	0.31 (0.09, 1.20); *n* = 28	*U* = 989	0.023	0.053
Group-by-time interaction				*F* = 13.222	<0.001	<0.001***
White blood cell count (×10⁹/L)						
T0	11.80 (8.25, 16.45); *n* = 103	13.05 (8.38, 18.18); *n* = 68	10.80 (8.00, 12.75); *n* = 35	*U* = 1382	0.184	0.259
T1	10.10 (7.40, 14.20); *n* = 101	10.70 (7.55, 15.35); *n* = 67	9.25 (6.95, 11.95); *n* = 34	*U* = 1310	0.219	0.259
T2	10.20 (6.55, 14.35); *n* = 83	11.35 (6.25, 15.67); *n* = 54	8.90 (6.60, 11.90); *n* = 29	*U* = 910	0.229	0.259
Group-by-time interaction				*F* = 0.225	0.799	0.799
Serum amyloid A (mg/L)^a^						
T0	200.00 (55.61, 311.43); *n* = 103	200.00 (168.58, 320.00); *n* = 68	57.31 (16.42, 158.55); *n* = 35	*U* = 1763	<0.001	<0.001***
T1	200.00 (109.46, 200.00); *n* = 101	200.00 (112.06, 201.43); *n* = 67	200.00 (101.80, 200.00); *n* = 34	*U* = 1300	0.237	0.259*
T2	130.89 (65.22, 200.00); *n* = 83	125.44 (61.32, 200.00); *n* = 54	139.23 (66.07, 200.00); *n* = 29	*U* = 804	0.844	0.844
Group-by-time interaction				*F* = 7.703	<0.001	<0.001***
Inflammatory cytokines at enrolment						
IL-2 (pg/mL)	57.55 (33.03, 66.71); *n* = 103	56.94 (33.10, 64.00); *n* = 68	62.81 (33.03, 69.27); *n* = 35	*U* = 957	0.105	0.132
IL-4 (pg/mL)	6.10 (2.91, 16.50); *n* = 102	7.69 (3.23, 20.48); *n* = 68	5.72 (2.67, 9.19); *n* = 34	*U* = 1338	0.198	0.198
IL-6 (pg/mL)	39.57 (15.11, 108.71); *n* = 103	65.22 (27.60, 193.73); *n* = 68	14.63 (7.06, 28.05); *n* = 35	*U* = 1926	<0.001	<0.001***
IL-10 (pg/mL)	9.88 (7.23, 23.80); *n* = 103	13.99 (7.74, 32.54); *n* = 68	8.21 (6.73, 10.63); *n* = 35	*U* = 1589	0.006	0.009**
TNF-α (pg/mL)	117.32 (88.54, 168.15); *n* = 103	143.40 (90.65, 179.33); *n* = 68	96.62 (76.93, 119.78); *n* = 35	*U* = 1627	0.002	0.006**
Circulating biomarkers of intestinal barrier dysfunction						
DAO (pg/mL)	6.72 (4.74, 9.23); *n* = 103	6.46 (4.71, 8.65); *n* = 68	7.66 (5.06, 9.66); *n* = 35	*U* = 1062	0.375	0.375
LBP (pg/mL)	16.61 (10.20, 24.22); *n* = 103	18.79 (12.14, 24.81); *n* = 68	12.46 (8.28, 18.24); *n* = 35	*U* = 1468	0.053	0.135
Zonulin (pg/mL)	1.62 (1.43, 3.25); *n* = 103	1.69 (1.47, 4.04); *n* = 68	1.49 (1.39, 2.01); *n* = 35	*U* = 1434	0.090	0.135

Data are presented as median (P25, P75), followed by the number of available observations. Missing values were not imputed.

T0 was enrolment, T1 was days 1–3 after enrolment and T2 was days 3–7 after enrolment. IL-2, IL-4, IL-6, IL-10, TNF-α, DAO, LBP and zonulin were measured only at T0.

Between-group comparisons used two-sided Mann–Whitney *U* tests. Group-by-time interactions used random-intercept linear mixed-effects models with time treated as categorical; CRP, PCT and SAA were log1p transformed and WBC was analysed on its original scale.

Benjamini-Hochberg correction was applied separately to conventional-marker pointwise comparisons, longitudinal interaction tests, baseline inflammatory cytokines and intestinal barrier biomarkers.

BH, Benjamini-Hochberg; CRP, C-reactive protein; DAO, diamine oxidase; IL, interleukin; LBP, lipopolysaccharide-binding protein; P25, 25th percentile; P75, 75th percentile; PCT, procalcitonin; SAA, serum amyloid A; TNF, tumour necrosis factor; WBC, white blood cell count.

a The upper reportable limit for SAA was 350 mg/L. Sixteen of 287 available T0–T2 measurements (5.6%) reached this limit: 15/103 at T0, 0/101 at T1 and 1/83 at T2; no measurement exceeded 350 mg/L.

* Adjusted *P* < 0.05.

** Adjusted *P* < 0.01.

*** Adjusted *P* < 0.001.

### 3.2 Gut microbial diversity and differential taxonomic features

Sepsis was associated with lower Shannon diversity (standardized *β* = −0.60, 95% CI −1.08 to −0.12, *P* = 0.0151, *q* = 0.0252; Figure 1a), Simpson diversity (*β* = −0.54, 95% CI −0.96 to −0.12, *P* = 0.014, *q* = 0.025; Supplementary Figure S1b) and Pielou evenness (*β* = −0.60, 95% CI −1.04 to −0.16, *P* = 0.008, *q* = 0.025; Supplementary Figure S1c). The estimates for Chao1 richness (*β* = −0.38, 95% CI −0.88 to 0.12, *q* = 0.136; Figure 1b) and observed species (*β* = −0.38, 95% CI −0.88 to 0.12, *q* = 0.136; Supplementary Figure S1a) were also negative. Negative effect estimates were observed across all prespecified models for each of the five alpha-diversity metrics (Supplementary Figure S2).

**Figure 1:**
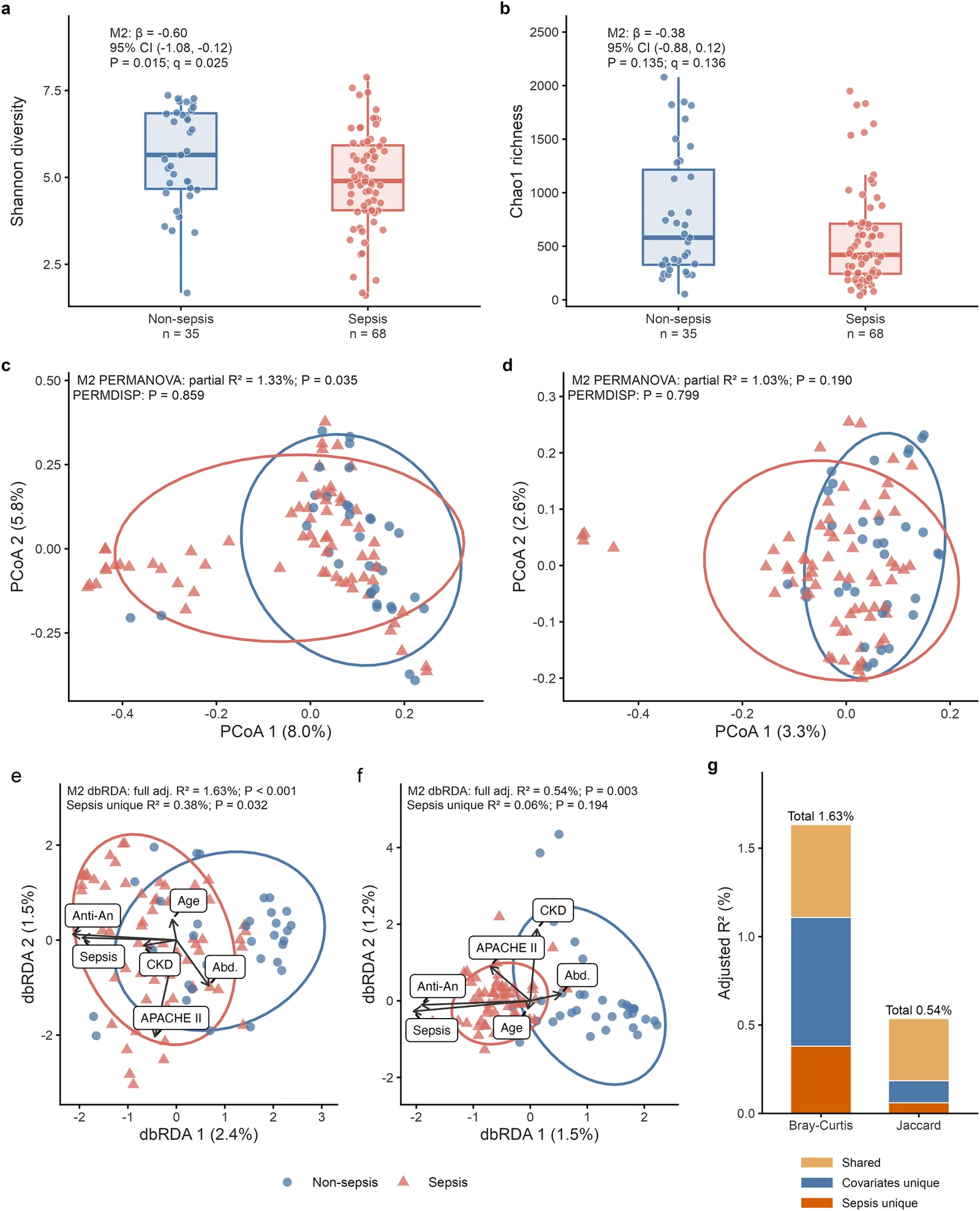
Gut microbial adjusted alpha- and beta-diversity comparisons in critically ill patients. **(a,b)** show the distributions of Shannon diversity and Chao1 richness, respectively; boxes indicate medians and interquartile ranges, whiskers extend to 1.5 times the interquartile range, and points represent individual patients. Standardized *β* coefficients compare sepsis with non-sepsis under M2, adjusted for age, intra-abdominal infection, pre-sampling anti-anaerobic antibiotic exposure, APACHE II score, and chronic kidney disease. Shannon diversity was standardized on its original scale, whereas Chao1 richness was log-transformed before standardization. The 95% confidence intervals and *P* values were calculated using HC3 heteroscedasticity-consistent standard errors. The *q* values were obtained using the Benjamini-Hochberg procedure across the five prespecified alpha-diversity metrics. **(c,d)** Principal coordinate analyses of Bray-Curtis and Jaccard dissimilarities after Lingoes correction. Points represent patients and ellipses are 80% group data ellipses; coordinates are unadjusted. **(e,f)** M2 distance-based redundancy analyses for Bray-Curtis and Jaccard dissimilarities. Arrows represent fitted constraints. Permutational *P* values used 9,999 permutations. **(g)** Partitioning of adjusted variance into sepsis-unique, covariate-unique, and shared fractions. APACHE II, Acute Physiology and Chronic Health Evaluation II; CI, confidence interval; dbRDA, distance-based redundancy analysis; PCoA, principal coordinate analysis; PERMANOVA, permutational multivariate analysis of variance; PERMDISP, permutational analysis of multivariate dispersion.

The Bray-Curtis and Jaccard PCoA ordinations showed considerable overlap between groups (Figures 1c, d). In M2-adjusted PERMANOVA, sepsis status accounted for 1.33% of Bray-Curtis variation (*P* = 0.0347, *q* = 0.0567; Figure 1c) and 1.03% of Jaccard variation (*P* = 0.1902, *q* = 0.1902; Figure 1d). The corresponding PERMDISP P values were 0.859 and 0.799. Weighted and unweighted UniFrac analyses yielded partial *R*^2^ values of 1.61% and 1.33%, respectively, with *q* = 0.0567 for both comparisons (Supplementary Figure S3a, b). Across all models and distance measures, partial *R*^2^ values ranged from 1.03% to 2.44% (Supplementary Figure S4), and all PERMDISP *P* values exceeded 0.05 (Supplementary Figure S5).

The full M2 covariate-adjusted dbRDA model explained 1.63% of Bray-Curtis variation (*P* < 0.001), including a 0.38% unique contribution from sepsis status (*P* = 0.032, *q* = 0.064; Figure 1e). For Jaccard dissimilarity, the full model explained 0.54% of variation (*P* = 0.0026), with a 0.06% unique contribution from sepsis status (*P* = 0.194, *q* = 0.194; Figure 1f). The unique covariate and shared components accounted for 0.73% and 0.53% of Bray-Curtis variation, and 0.12% and 0.35% of Jaccard variation, respectively (Figure 1g). The variance partitioning thus showed that the full M2 model, including sepsis status and all covariates, explained only 1.63% and 0.54% of the Bray-Curtis and Jaccard variation, respectively, leaving more than 98% of the community variation unexplained.

In the primary genus-level discovery analysis, four of 190 genera met the Wilcoxon criteria of *q* < 0.05 and |log_2_ fold change| > 1 (Figure 2a). *Peptoniphilus_B* (log_2_ fold change = −4.75, *q* = 0.0216), *Fenollaria* (−2.99, *q* = 0.0216) and *Ezakiella* (−1.60, *q* = 0.0401) were less abundant in Sepsis, whereas *Lactococcus_A* was more abundant (3.00, *q* = 0.0401). These four taxa are referred to as the Wilcoxon-identified genera and were carried forward for exploratory clinical-association analyses.

**Figure 2:**
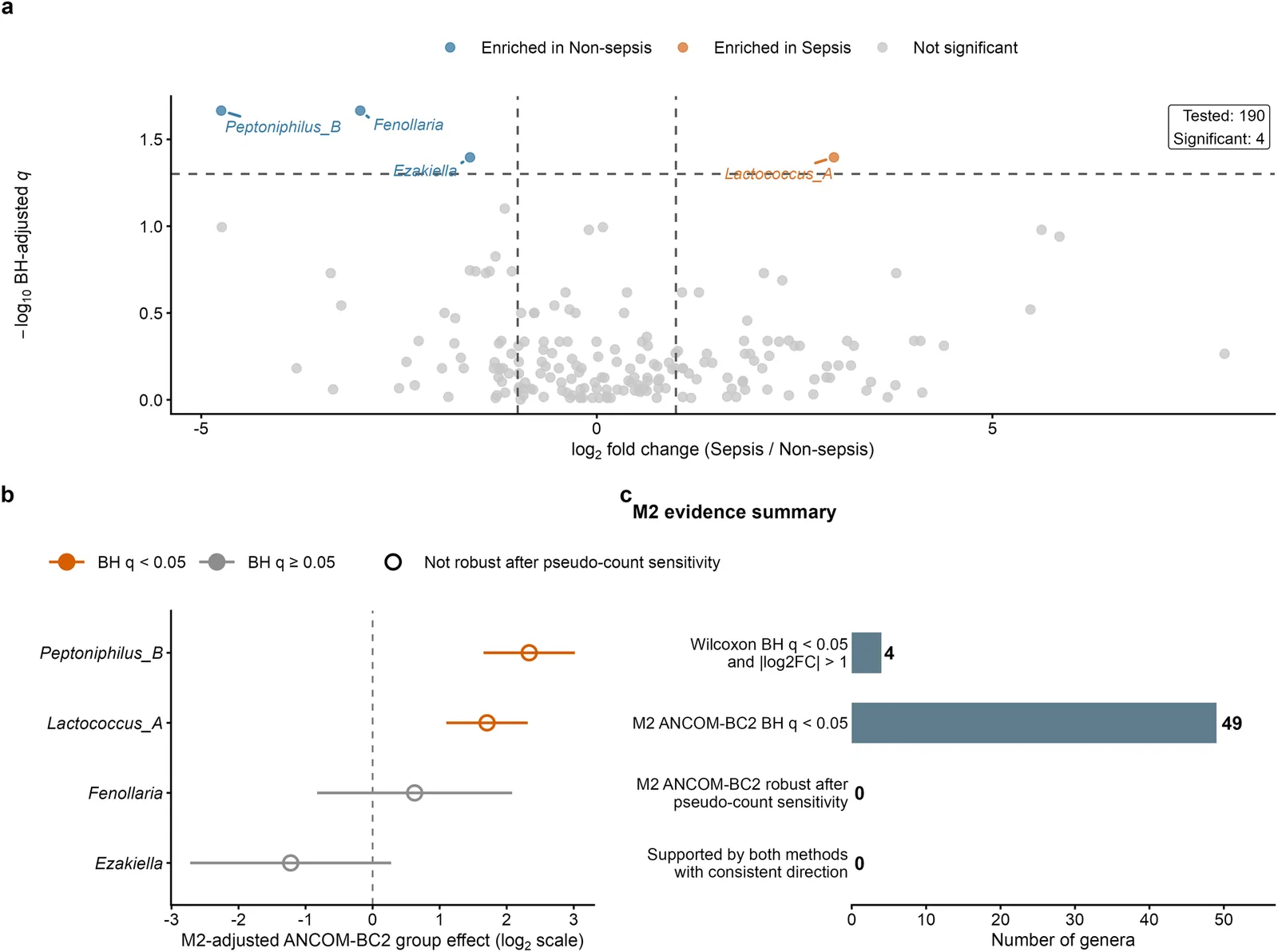
Genus-level differential abundance analysis of the gut microbiota in critically ill patients with and without sepsis. **(a)** Wilcoxon rank-sum comparisons of 190 genera between the sepsis and non-sepsis groups. The axes show the log_2_ fold change for Sepsis relative to non-sepsis and −log_10_ of the Benjamini-Hochberg-adjusted *q* value. Dashed lines indicate |log_2_ fold change| = 1 and *q* = 0.05. Four genera met both criteria. Blue and orange indicate lower and higher relative abundance in Sepsis, respectively; grey indicates genera that did not meet both criteria. **(b)** M2-adjusted ANCOM-BC2 log_2_ effect estimates and 95% confidence intervals for the four Wilcoxon candidates. M2 adjusted for age, intra-abdominal infection, documented pre-sampling anti-anaerobic exposure, APACHE II score and chronic kidney disease. Orange and grey indicate *q* < 0.05 and *q* ≥ 0.05; open circles indicate results that were not robust to pseudo-count sensitivity analysis. **(c)** Four genera met the Wilcoxon criteria, 49 of 190 tested genera met the M2-adjusted ANCOM-BC2 *q* < 0.05 criterion, none remained robust after pseudo-count sensitivity analysis, and none was supported by both methods with a consistent direction. BH correction was applied separately across all 190 genera within each method.

M2-adjusted ANCOM-BC2 yielded *q* < 0.05 for 49 genera (Figure 2c). Among the four Wilcoxon-identified genera, *Lactococcus_A* (log_2_ effect = 1.71, 95% CI 1.10 to 2.31, *q* = 1.18 × 10^−4^) and *Peptoniphilus_B* (2.34, 95% CI 1.66 to 3.02, *q* = 7.77 × 10^−5^) were significant (Figure 2b). *Ezakiella* (−1.22, 95% CI −2.72 to 0.28, *q* = 0.269) and *Fenollaria* (0.63, 95% CI −0.83 to 2.08, *q* = 0.635) were not significant (Figure 2b). The adjusted direction for *Peptoniphilus_B* was opposite to its Wilcoxon estimate. After pseudo-count sensitivity testing, the number of robust ANCOM-BC2 genera was zero in M2 and the other prespecified models (Figure 2c; Supplementary Figures S6 and S7).

In the complementary fully adjusted MaAsLin2 M2 sensitivity analysis, no genus reached significance (minimum *q* = 0.333; Supplementary Figure S8). Together with the lack of robust ANCOM-BC2 findings after pseudo-count sensitivity testing, this result indicated that the four Wilcoxon-identified genera did not receive consistent support across analytical methods.

### 3.3 Clinical associations of the Wilcoxon-identified genera

Adjusted partial correlations between the four Wilcoxon-identified genera and baseline inflammatory or intestinal-barrier markers ranged from −0.13 to 0.20; none reached *q* < 0.05 after domain-specific FDR correction (Figure 3a). No statistically significant associations were observed for ARDS, AKI or AGI after correction within the major-complication domain (Figure 3b). *Peptoniphilus_B* showed the strongest associations with mortality. Higher abundance corresponded to lower odds of 28-day mortality (adjusted OR = 0.52 per 1-s.d. increase in abundance rank, 95% CI 0.31–0.89, *P* = 0.0165, *q* = 0.0661) and in-hospital mortality (adjusted OR = 0.48, 95% CI 0.28–0.83, *P* = 0.0081, *q* = 0.0646; Figure 3c). However, as discussed below, these estimates may be subject to residual confounding from unmeasured factors including proton pump inhibitor use, detailed nutritional regimens, and organ-support modalities, and should therefore be interpreted with considerable caution. In sensitivity analyses, *Peptoniphilus_B* was associated with both mortality outcomes in M0 and M1, while *Ezakiella* was associated with in-hospital mortality in these models. No mortality association reached *q* < 0.05 in M2, S1, S2 or R1 (Supplementary Figure S9). Associations with ICU stay, hospital stay, ventilator days and vasoactive medication days were non-significant after correction (Figure 3d).

**Figure 3:**
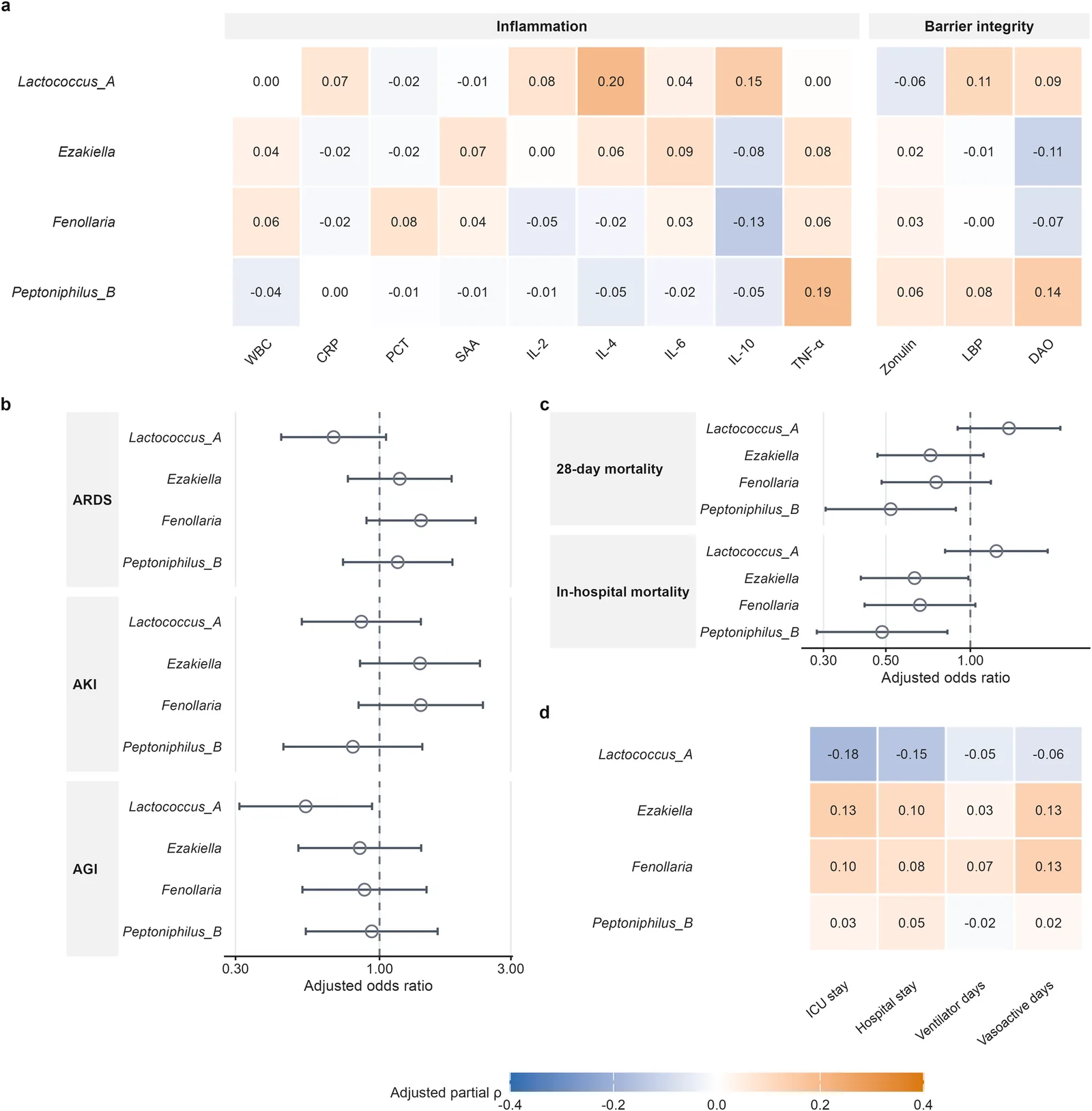
Associations of Wilcoxon-identified genera with clinical characteristics and outcomes in critically ill patients. All analyses used the primary M2 model. **(a)** Adjusted partial Spearman correlations with baseline inflammatory markers and circulating markers of intestinal barrier integrity. **(b)** Adjusted odds ratios for ARDS, AKI and AGI. **(c)** Adjusted odds ratios for 28-day and in-hospital mortality. **(d)** Adjusted partial Spearman correlations with ICU stay, hospital stay, ventilator days and vasoactive medication days. Heatmap cells show adjusted partial Spearman *ρ* values. Odds ratios are expressed per 1-s.d. increase in genus relative-abundance rank, with horizontal lines indicating 95% confidence intervals. Filled blue and open grey points denote *q* < 0.05 and *q* ≥ 0.05, respectively. Asterisks indicate Benjamini-Hochberg-adjusted significance thresholds: *q < 0.05, **q < 0.01 and ***q < 0.001. Benjamini-Hochberg correction was applied separately within each prespecified clinical domain. AGI, acute gastrointestinal injury; AKI, acute kidney injury; APACHE II, Acute Physiology and Chronic Health Evaluation II; ARDS, acute respiratory distress syndrome; BH, Benjamini-Hochberg; CI, confidence interval; CKD, chronic kidney disease; FDR, false discovery rate; ICU, intensive care unit.

In longitudinal linear mixed-effects models treating genus abundance as a continuous variable, we identified three significant time-by-genus interactions among the 12 prespecified genus-marker combinations. Primary models included 102 participants, contributing 285 PCT and 286 CRP or WBC observations. Figure 4 illustrates these associations using model-derived P75-P25 contrasts. *Fenollaria* abundance showed a significant interaction with the longitudinal CRP profile (*P* = 0.002, *q* = 0.010; Figure 4c). *Peptoniphilus_B* abundance was associated with the longitudinal profiles of PCT (*P* = 0.0104 *q* = 0.042) and CRP (*P* = 0.002, *q* = 0.010; Figure 4d). No significant interactions were observed for *Lactococcus_A* (Figure 4a), *Ezakiella* (Figure 4b) or any WBC profile.

**Figure 4:**
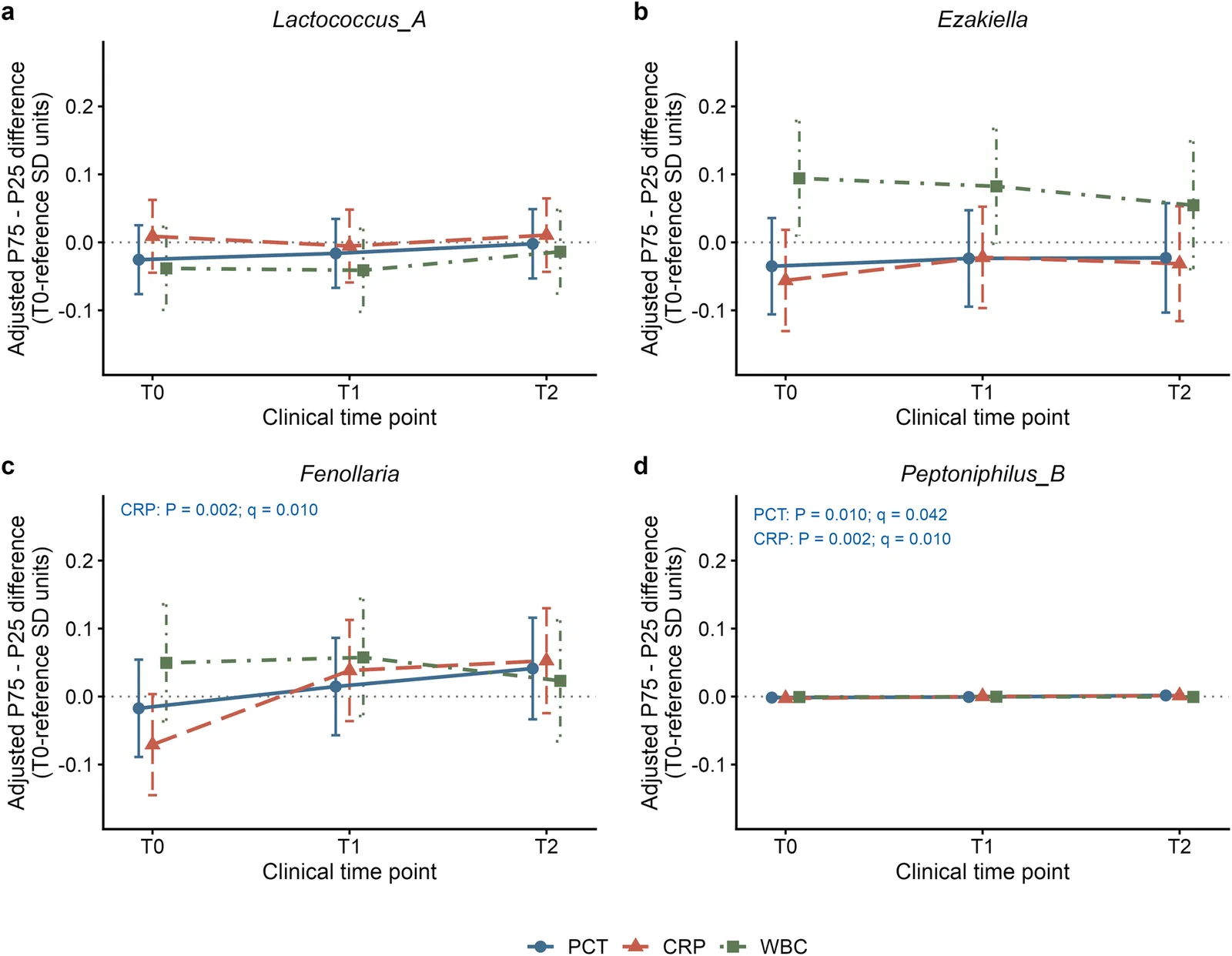
Associations of baseline Wilcoxon-identified genera with longitudinal inflammatory profiles in critically ill patients. **(a–d)** Associations of baseline *Lactococcus_A, Ezakiella, Fenollaria* and *Peptoniphilus_B* abundances, respectively, with PCT, CRP and WBC from T0 to T2. Points and lines show adjusted P75–P25 differences; error bars indicate 95% confidence intervals. Positive values indicate higher estimated marker levels at P75. Genus abundance was log1p-transformed, standardized and analysed continuously; P25 and P75 were used only for visualization. PCT and CRP were log1p-transformed, whereas WBC was untransformed. Outcomes are expressed in pooled T0-standard-deviation units. M2 adjusted for age, intra-abdominal infection, documented pre-sampling anti-anaerobic exposure, APACHE II and chronic kidney disease. *P* values are two-degree-of-freedom likelihood-ratio tests of the overall time-by-genus interaction; *q* values were Benjamini-Hochberg-adjusted across 12 prespecified tests. Only *q* < 0.05 results are annotated. CI, confidence interval; CRP, C-reactive protein; P25/P75, 25th/75th percentile; PCT, procalcitonin; WBC, white blood cell count.

The *Fenollaria*–CRP interaction reached *q* < 0.05 in seven of nine analysis sets, including M0, M1, M2, S1, S2, R1 and the complete-trajectory analysis. The *Peptoniphilus_B*–CRP interaction reached *q* < 0.05 in six analysis sets, whereas its PCT interaction was significant only in M2 and S1 (Supplementary Figure S10). Neither the group-by-time nor the detection sensitivity analysis yielded an interaction with *q* < 0.05.

## 4 Discussion

This study identified a conditional microbiome signal among critically ill patients with and without sepsis. After full adjustment, selected alpha-diversity and evenness measures were lower in the Sepsis group, whereas richness measures and overall community composition showed limited between-group differences. Wilcoxon screening identified higher *Lactococcus_A* and lower *Ezakiella*, *Fenollaria* and *Peptoniphilus_B* abundances in Sepsis, but these signals were not consistently confirmed by ANCOM-BC2 sensitivity testing or MaAsLin2. Associations with baseline inflammatory markers, intestinal-barrier-related markers, major complications and clinical outcomes were limited. Nevertheless, longitudinal associations of *Fenollaria* and *Peptoniphilus_B* with inflammatory-marker profiles suggest a potential time-dependent relationship between baseline microbial composition and subsequent systemic inflammation.

In the fully adjusted M2 model, Shannon diversity, Simpson diversity and Pielou evenness were significantly lower in the Sepsis group after FDR correction, whereas Chao1 richness and observed species were not. This pattern suggests that sepsis-related differences were more evident in community diversity and evenness than in richness. Reduced alpha diversity has been reported in critically ill patients compared with healthy controls (4), whereas evidence for an additional sepsis-specific reduction among critically ill patients is inconsistent and may depend on sampling time and antimicrobial exposure (4, 6). Differences in sampling time, antimicrobial exposure, case mix and analytical adjustment may contribute to this heterogeneity, although the contribution of any individual factor cannot be determined from the present models.

Beta-diversity analyses suggested, at most, a small and distance-metric-dependent difference in community composition, without clear separation between groups. In the M2 PERMANOVA, the Bray-Curtis group effect was nominally significant (partial *R*^2^ = 1.33%, *P* = 0.0347) but did not meet the FDR threshold (*q* = 0.0567). Jaccard dissimilarity was not significant (partial *R*^2^ = 1.03%, *P* = 0.1902, *q* = 0.1902), whereas both UniFrac metrics produced similarly borderline results (*q* = 0.0567). PERMDISP provided no evidence that unequal within-group dispersion accounted for these findings. Although the overall dbRDA models were significant, neither group-specific fraction remained significant after FDR correction. Group status uniquely explained only 0.38% of Bray-Curtis and 0.06% of Jaccard variation, with more than 98% unexplained by the variables included in M2. Together with the overlapping PCoA ordinations, these findings indicate that any sepsis-associated compositional signal was small relative to marked interindividual heterogeneity. This heterogeneity is consistent with the profound microbiota disruption and occasional taxon domination described during critical illness (5). Sepsis-related differences may also depend on sampling time and antimicrobial exposure, as one cohort detected differences during early but not later treatment periods (6).

In this study, the three differential-abundance methods we employed did not yield convergent results. The Wilcoxon rank-sum test was selected as the primary discovery tool because it directly compares skewed relative abundance distributions without a normality assumption, and, as some benchmark studies suggest, nonparametric approaches can offer favorable FDR control and replicability in certain settings (7–9). However, a limitation of such univariate tests is that they do not account for covariates or the compositional nature of microbiome data. Therefore, we applied MaAsLin2 for covariate-adjusted analysis and ANCOM-BC2 with pseudo-count sensitivity testing for compositional analysis, to assess the robustness of our findings. In our cohort, the Wilcoxon test identified four genera, but these signals were not consistently supported by the complementary methods: the ANCOM-BC2 signals were not robust to pseudo-count sensitivity testing, and MaAsLin2 identified no FDR-significant genera. This discordance is not unexpected. Previous benchmark studies have demonstrated substantial disagreement among differential-abundance methods, highlighting that results are sensitive to analytical choices such as zero handling and data transformation (7–9). The lack of cross-method concordance suggests that our Wilcoxon-identified genera should be interpreted as exploratory hypotheses rather than as methodologically robust, replicated taxa.

The lower Wilcoxon estimates for *Ezakiella* and *Peptoniphilus_B* broadly agree with an earlier ICU study reporting reduced abundances of these genera in sepsis compared with non-septic critical illness (10). Another longitudinal sepsis cohort documented time-dependent changes in *Ezakiella* and *Peptoniphilus* and their relationships with clinical variables (11). However, the four Wilcoxon-identified genera showed no consistent associations with baseline inflammatory markers or major clinical outcomes in our cohort. Longitudinal analyses linked baseline *Fenollaria* abundance to subsequent CRP trajectories and *Peptoniphilus_B* abundance to PCT and CRP trajectories, suggesting that selected microbial differences may be related to changes in systemic inflammation during critical illness. These relationships are biologically plausible because gut microbial ecology, intestinal-barrier function and host inflammation may influence one another during sepsis (1), while antimicrobial exposure may further reshape anaerobic microbial communities (12). Because these specific genus–inflammation associations have not, to our knowledge, been previously reported, independent longitudinal cohorts is required.

The largely null and method-sensitive findings may reflect the extensive microbiome disturbance shared by both ICU groups. PERMDISP provided no evidence that unequal within-group dispersion explained the PERMANOVA results, and dbRDA attributed little community variation uniquely to sepsis. A prospective longitudinal ICU study documented progressive losses of microbial diversity and marked compositional changes during the first week of critical illness; microbiota composition was associated with the duration of antibiotic treatment before sampling but not with SOFA score or admission diagnosis (13). Temporal dynamics and antimicrobial exposure may therefore have obscured smaller sepsis-specific signals. Sparse genus-level distributions and method-specific assumptions may also have reduced estimate stability (7–9). Accordingly, the Wilcoxon-identified genera should be interpreted cautiously and assessed with multiple complementary methods. Although M2 included pre-sampling anti-anaerobic exposure, antibiotic class, exact timing, duration and dose were not modelled at the genus level; the enrichment of *Lactococcus_A* therefore cannot be attributed specifically to antibiotic selection. Nevertheless, antibiotic exposure remains a plausible contributor because anti-anaerobic antibiotics can disproportionately disrupt the ICU gut microbiota (12).

The intestinal-barrier findings require separate consideration because their interpretation depends on biomarker validity. In a human validation study, commercial zonulin ELISA measurements neither agreed with pre-haptoglobin-2 nor reliably reflected colonic paracellular permeability (14). The zonulin results in our cohort therefore provide limited information about intestinal permeability. I-FABP and REG3α may provide complementary measures of intestinal-wall injury in critically ill patients, but neither was measured in this study (15).

The present findings support a longitudinal and exposure-aware approach to studying microbiome-inflammation relationships. Building on ICU studies that combined serial microbiome sampling with immune and inflammatory profiling (13), future cohorts should incorporate repeated SOFA assessments to characterize microbial, inflammatory and organ-dysfunction trajectories. Antibiotic class, treatment timing, cumulative anti-anaerobic exposure, enteral formula, fibre content and energy delivery should be recorded prospectively and modelled as time-varying exposures. Because habitual diet and geographical context shape gut microbiome composition, multicentre studies should capture these factors when assessing between-cohort heterogeneity (16). Differences in ICU practice could be evaluated concurrently as potential modifiers of microbiome-host relationships.

Mechanistic studies could incorporate I-FABP and REG3α, which have been evaluated as candidate markers of intestinal-wall injury in patients with MODS (15), together with direct measures of permeability and microbial translocation. Integrating these measurements with shotgun metagenomics and metabolomics could link taxonomic variation to intestinal injury, microbial function and systemic inflammation. The associations between baseline *Fenollaria* and *Peptoniphilus_B* abundances and subsequent inflammatory-marker trajectories provide specific hypotheses for further investigation. Serial microbiome and metabolomic profiling could determine whether these signals coincide with changes in SCFA metabolism and systemic inflammation. A recent phase 2 trial found that inulin did not alter SCFA-producing bacteria, microbial diversity or clinical outcomes in ICU patients with sepsis (17). Future microbiome-directed nutritional trials should therefore use clearly defined biological targets, mechanistically relevant endpoints and prespecified patient populations.

Several limitations should be considered. The single-centre design and sample size limit generalisability and statistical precision, particularly after FDR correction and for outcomes with limited event counts. Centre-specific differences in antibiotic stewardship, infection sources, organ-support practices, dietary backgrounds and nutritional protocols may also have influenced microbial profiles. The Non-sepsis group consisted of critically ill patients rather than healthy participants. This provided a clinically relevant comparison but did not quantify the overall effect of critical illness. Our findings should be interpreted in light of several limitations, including the potential for residual confounding. Although M2 adjusted for age, intra-abdominal infection, documented pre-sampling anti-anaerobic exposure, APACHE II score and chronic kidney disease, several important potential confounders were not recorded, which could have substantially influenced the observed microbial associations. Prominent among these are proton pump inhibitor or H2-receptor antagonist use, detailed nutritional regimens (including enteral formula composition, fibre content, energy delivery, and their temporal relationship to sampling), and time-resolved organ-support modalities. Their absence from our models means that the adjusted associations we report, particularly the mortality associations for *Peptoniphilus_B*, should be interpreted with considerable caution, as residual confounding from these unmeasured factors cannot be excluded. These unmeasured factors are particularly relevant when interpreting the adjusted associations reported in our primary M2 models and sensitivity analyses, as they may have contributed to residual confounding beyond the covariates we were able to include. Although nutritional route at the time of microbiota sampling was documented, the specific nutritional regimen, its exact timing and temporal relationship to sampling, together with enteral formula, fibre content and energy delivery, were not recorded in sufficient detail to assess nutritional effects on the gut microbiota. No genus-specific signal, including *Lactococcus_A* enrichment, can therefore be attributed to a particular antibiotic exposure.

The use of 16S rRNA sequencing limited taxonomic and functional resolution, and microbial metabolites were not measured. Although PCR-negative controls were included, they were not sequenced through the complete workflow; blank-swab and DNA-extraction controls were not sequenced, and bacterial load was not independently quantified. Potential low-biomass contamination and absolute bacterial abundance could therefore not be assessed retrospectively. In addition, the exact numerical DADA2 denoising parameters were not retained, preventing full reproduction of the initial read-processing step. Future independent cohorts with fully documented parameters and raw data are needed to confirm our observations.

Microbiota was assessed only at baseline, preventing evaluation of microbial trajectories. It was therefore not possible to determine whether baseline microbiota predicted longitudinal SOFA patterns. Cytokines and intestinal-barrier markers were measured only at T0, while conventional inflammatory markers were followed only to T2. DAO, LBP and zonulin are indirect circulating measures of intestinal injury. Commercial zonulin ELISAs may not specifically quantify pre-haptoglobin-2 or reliably reflect intestinal permeability (14), and complementary markers such as I-FABP and REG3α were not measured (15). Direct permeability and microbial-translocation measurements were also unavailable. Linear mixed-effects models used all available repeated measurements and accounted for within-participant correlation, but they do not eliminate bias when missingness is related to death or clinical deterioration. Consequently, estimates at later time points may remain affected by informative dropout and survivor selection.

Finally, the genus-level findings were sensitive to analytical assumptions and all genus–clinical association results should be considered exploratory. Interpretation of adjusted estimates also remains subject to residual confounding from unmeasured proton-pump-inhibitor use, detailed nutritional route and fibre exposure, and organ-support modalities, as described above.

## 5 Conclusion

In summary, patients with sepsis exhibited more pronounced systemic inflammation and lower microbial diversity and evenness than non-septic critically ill patients, whereas differences in overall community composition were modest. The primary Wilcoxon analysis identified four genus-level differences, but these signals were not consistently supported by complementary ANCOM-BC2 and MaAsLin2 analyses. Their associations with baseline inflammatory and intestinal-barrier markers, major complications and clinical outcomes were limited. However, the time-dependent associations of *Fenollaria* with CRP and of *Peptoniphilus_B* with PCT and CRP suggest a possible relationship between baseline microbial composition and subsequent inflammatory trajectories. These results indicate a conditional and exploratory microbiome signal rather than a stable sepsis-specific microbial signature or an established prognostic mechanism. Because healthy controls were not included, the contribution of critical illness itself could not be distinguished from that of sepsis. Larger multicentre studies incorporating serial microbiome sampling, detailed treatment and nutritional exposures, higher-resolution microbiome profiling, microbial metabolites and direct measurements of intestinal permeability are required to confirm these observations. Such studies could use predefined microbial, inflammatory and clinical endpoints to evaluate SCFA-targeted nutritional strategies and anaerobe-sparing antimicrobial approaches.

## Data Availability

The raw data supporting the conclusions of this article will be made available by the authors, without undue reservation.
