# Supplementary material for: Gut microbiome diversity, genus-level variation, and associations with clinical features in critically ill patients
Source: Front Med (Lausanne). 2026 Sep 23;13:1903736. doi: 10.3389/fmed.2026.1903736 (PMC13642589; doi:10.3389/fmed.2026.1903736)
Supplement: Supplementary file 1 [file Image1.pdf]

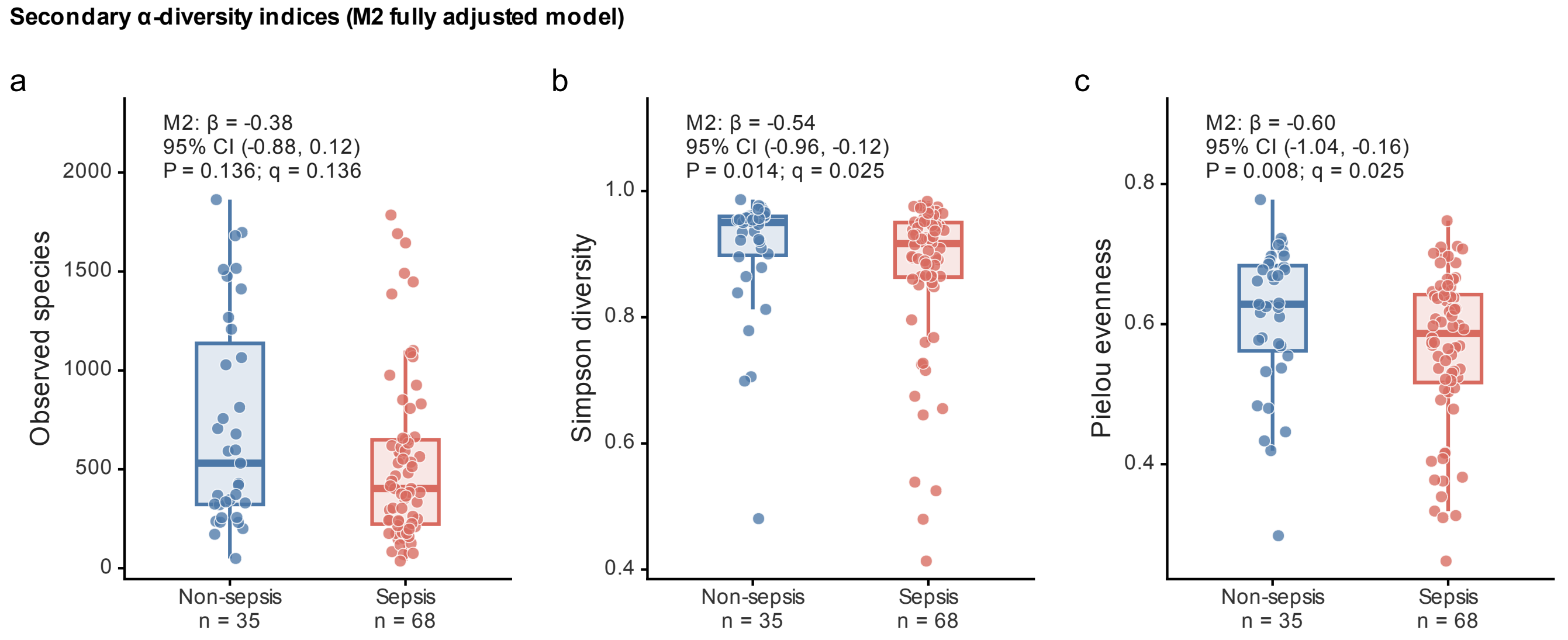
Secondary α-diversity indices (M2 fully adjusted model)
a
M2: β = -0.38
95% CI (-0.88, 0.12)
P = 0.136; q = 0.136
Observed species
0
500
1000
1500
2000
Non-sepsis
n = 35
Sepsis
n = 68
b
M2: β = -0.54
95% CI (-0.96, -0.12)
P = 0.014; q = 0.025
Simpson diversity
0.4
0.6
0.8
1.0
Non-sepsis
n = 35
Sepsis
n = 68
c
M2: β = -0.60
95% CI (-1.04, -0.16)
P = 0.008; q = 0.025
Pielou evenness
0.4
0.6
0.8
Non-sepsis
n = 35
Sepsis
n = 68
