# Supplementary material for: Gut microbiome diversity, genus-level variation, and associations with clinical features in critically ill patients
Source: Front Med (Lausanne). 2026 Sep 23;13:1903736. doi: 10.3389/fmed.2026.1903736 (PMC13642589; doi:10.3389/fmed.2026.1903736)
Supplement: Supplementary file 2 [file Image10.pdf]

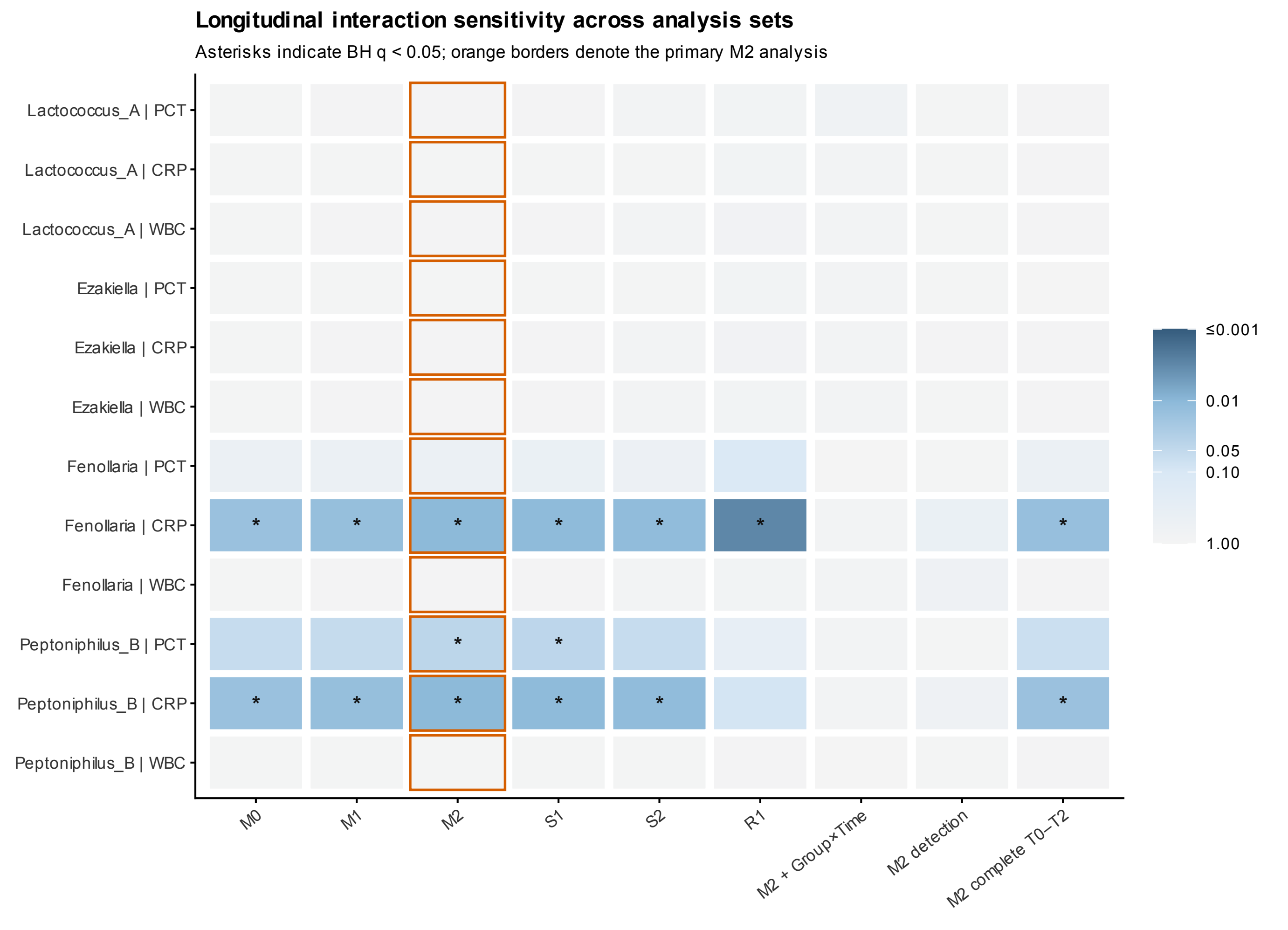

Longitudinal interaction sensitivity across analysis sets
Asterisks indicate BH q < 0.05; orange borders denote the primary M2 analysis
Lactococcus_A | PCT
Lactococcus_A | CRP
Lactococcus_A | WBC
Ezakiella | PCT
Ezakiella | CRP
Ezakiella | WBC
Fenollaria | PCT
Fenollaria | CRP
Fenollaria | WBC
Peptoniphilus_B | PCT
Peptoniphilus_B | CRP
Peptoniphilus_B | WBC
M0
M1
M2
S1
S2
R1
M2 + Group×Time
M2 detection
M2 complete T0–T2
≤0.001
0.01
0.05
0.10
1.00
