# Supplementary material for: Gut microbiome diversity, genus-level variation, and associations with clinical features in critically ill patients
Source: Front Med (Lausanne). 2026 Sep 23;13:1903736. doi: 10.3389/fmed.2026.1903736 (PMC13642589; doi:10.3389/fmed.2026.1903736)
Supplement: Supplementary file 3 [file Image2.pdf]

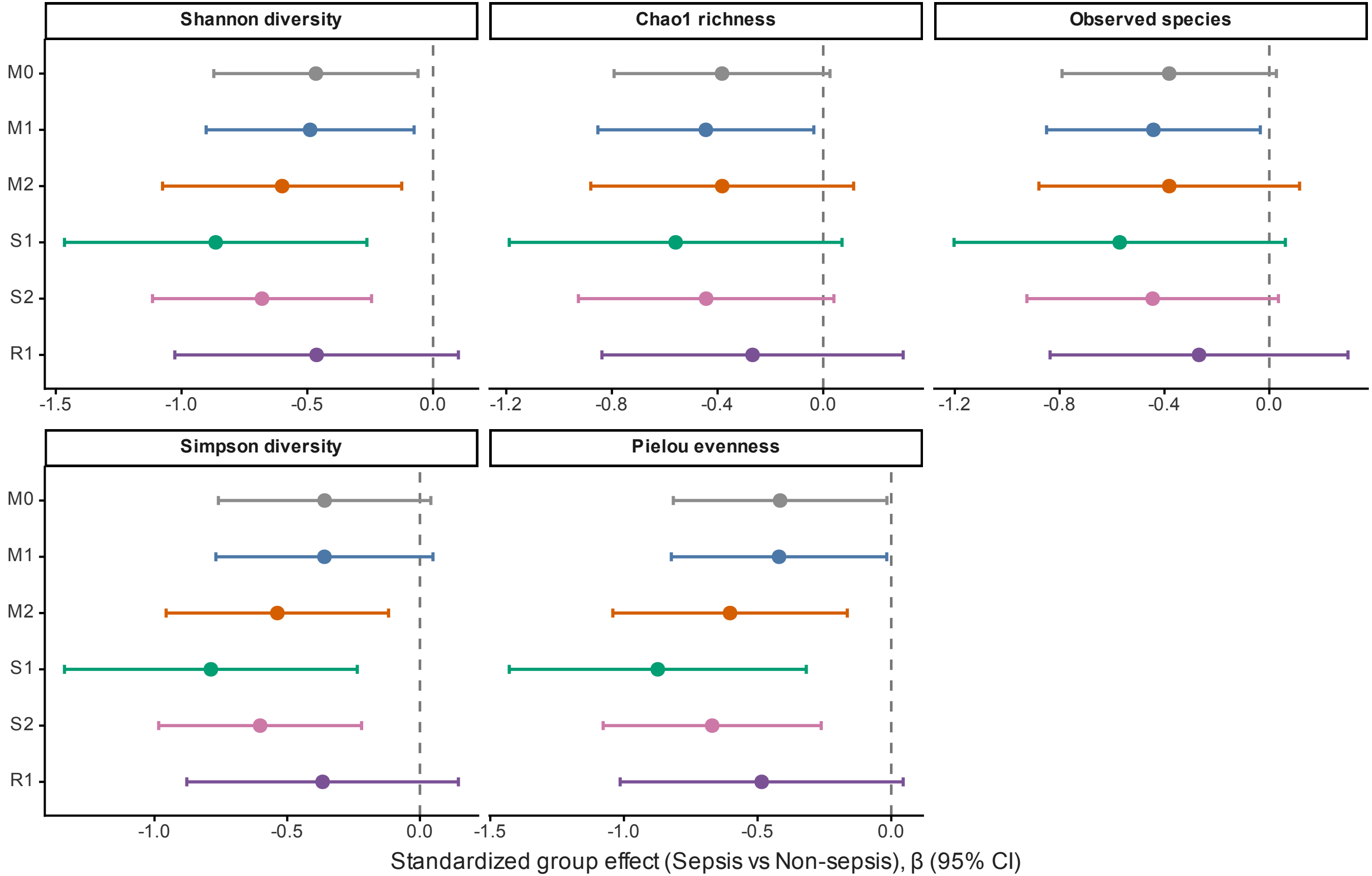

Shannon diversity
Chao1 richness
Observed species
Simpson diversity
Pielou evenness
M0
M1
M2
S1
S2
R1
-1.5
-1.0
-0.5
0.0
-1.2
-0.8
-0.4
0.0
-1.2
-0.8
-0.4
0.0
-1.0
-0.5
0.0
-1.5
-1.0
-0.5
0.0
Standardized group effect (Sepsis vs Non-sepsis), β (95% CI)
