# Supplementary material for: Gut microbiome diversity, genus-level variation, and associations with clinical features in critically ill patients
Source: Front Med (Lausanne). 2026 Sep 23;13:1903736. doi: 10.3389/fmed.2026.1903736 (PMC13642589; doi:10.3389/fmed.2026.1903736)
Supplement: Supplementary file 5 [file Image4.pdf]

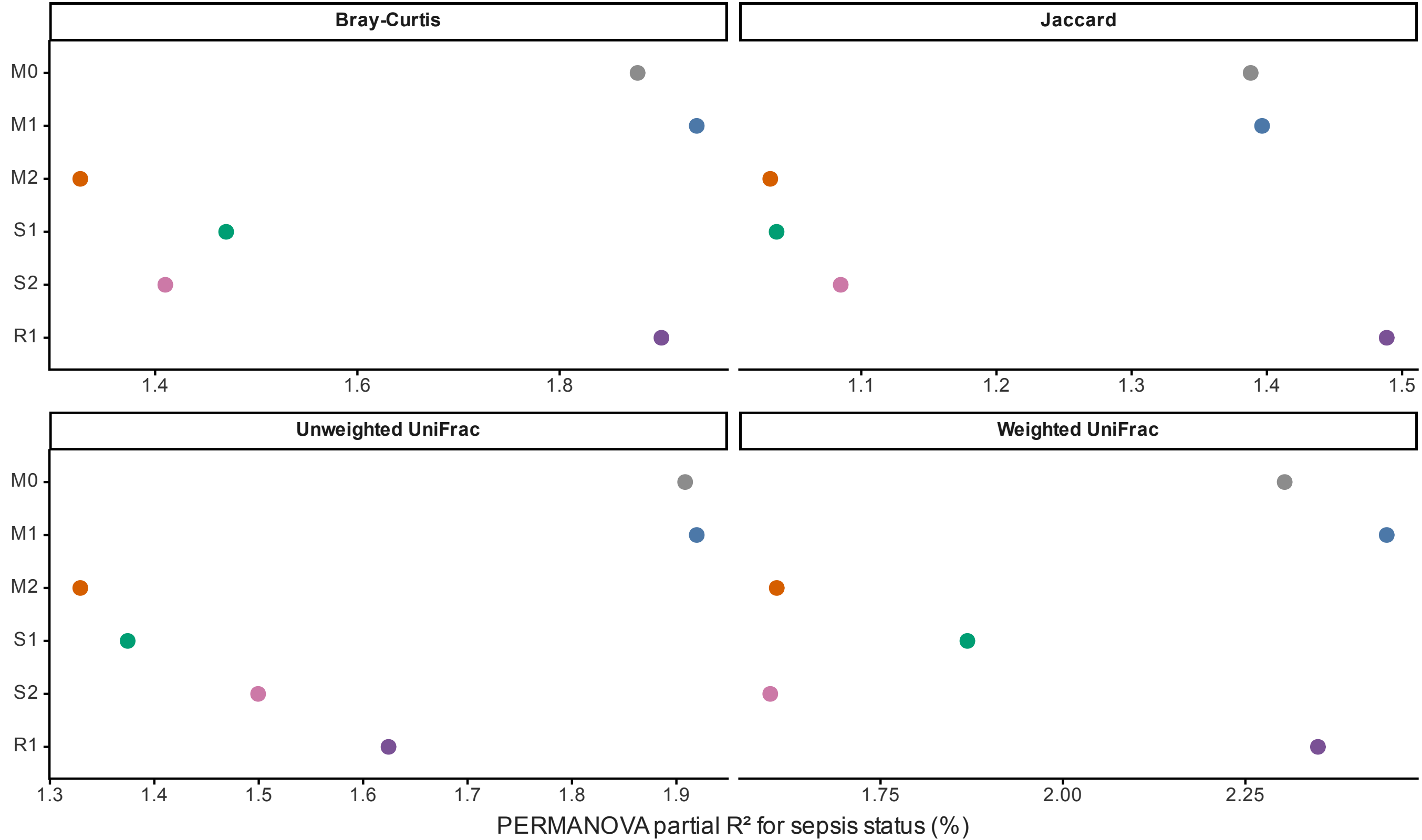
Bray-Curtis
Jaccard
Unweighted UniFrac
Weighted UniFrac
M0
M1
M2
S1
S2
R1
1.4
1.6
1.8
1.1
1.2
1.3
1.4
1.5
1.3
1.4
1.5
1.6
1.7
1.8
1.9
1.75
2.00
2.25
PERMANOVA partial R² for sepsis status (%)
