# Supplementary material for: Gut microbiome diversity, genus-level variation, and associations with clinical features in critically ill patients
Source: Front Med (Lausanne). 2026 Sep 23;13:1903736. doi: 10.3389/fmed.2026.1903736 (PMC13642589; doi:10.3389/fmed.2026.1903736)
Supplement: Supplementary file 6 [file Image5.pdf]

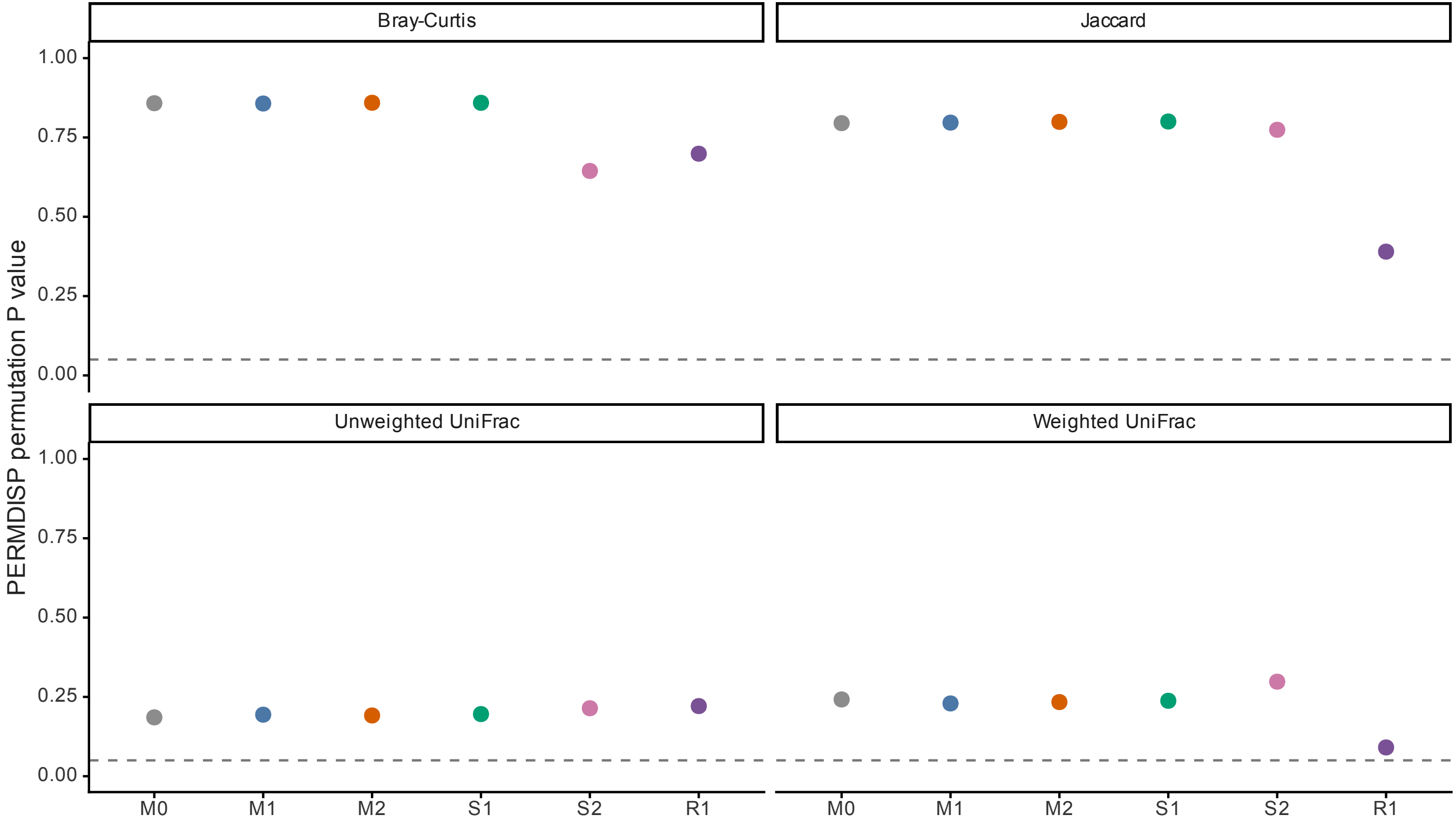
Bray-Curtis
Jaccard
Unweighted UniFrac
Weighted UniFrac
PERMDISP permutation P value
1.00
0.75
0.50
0.25
0.00
M0
M1
M2
S1
S2
R1
