# Supplementary material for: Gut microbiome diversity, genus-level variation, and associations with clinical features in critically ill patients
Source: Front Med (Lausanne). 2026 Sep 23;13:1903736. doi: 10.3389/fmed.2026.1903736 (PMC13642589; doi:10.3389/fmed.2026.1903736)
Supplement: Supplementary file 8 [file Image7.pdf]

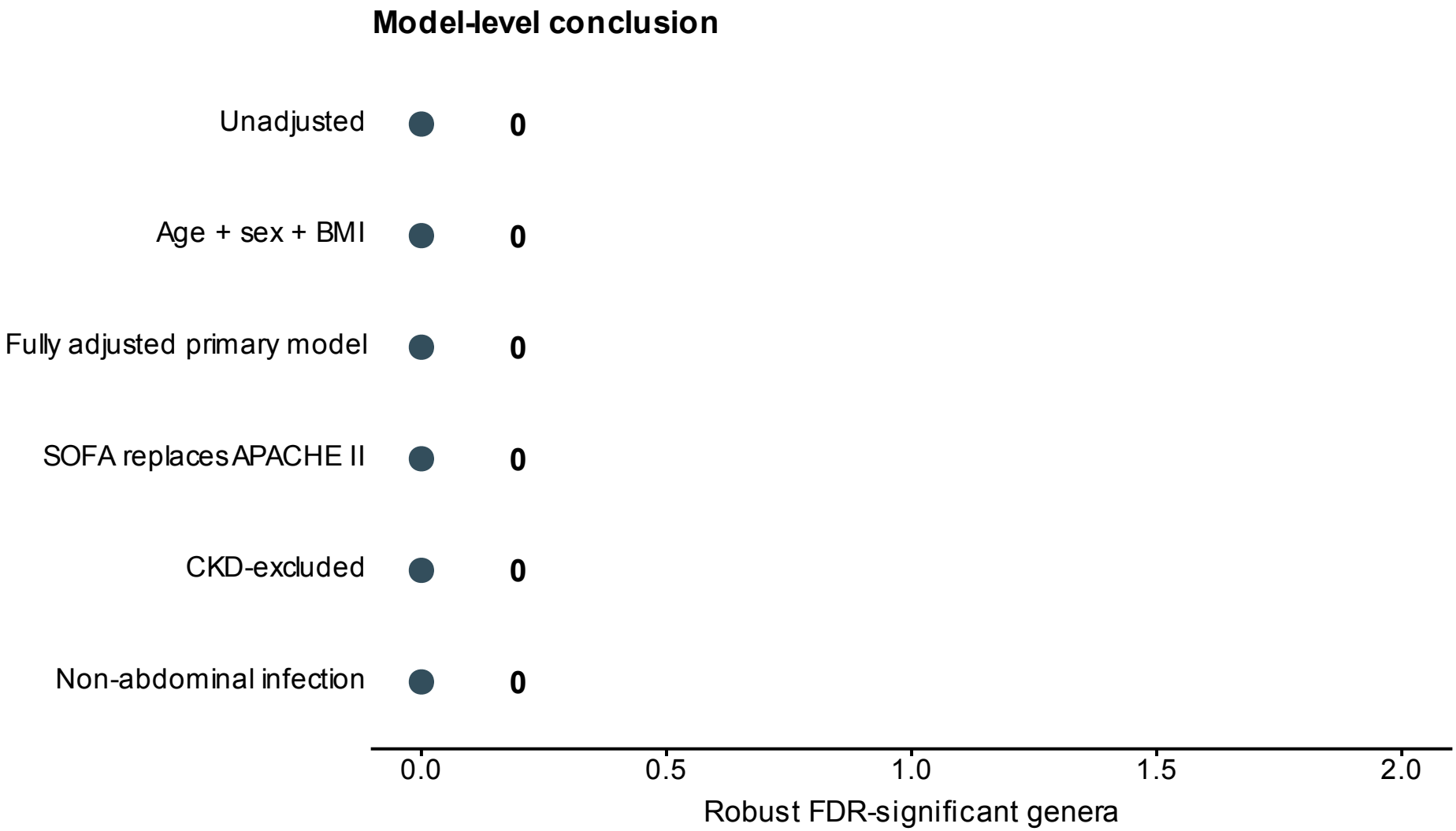
Model-level conclusion
Unadjusted
Age + sex + BMI
Fully adjusted primary model
SOFA replaces APACHE II
CKD-excluded
Non-abdominal infection
0
0
0
0
0
0
0.0
0.5
1.0
1.5
2.0
Robust FDR-significant genera
