# Supplementary material for: Gut microbiome diversity, genus-level variation, and associations with clinical features in critically ill patients
Source: Front Med (Lausanne). 2026 Sep 23;13:1903736. doi: 10.3389/fmed.2026.1903736 (PMC13642589; doi:10.3389/fmed.2026.1903736)
Supplement: Supplementary file 9 [file Image8.pdf]

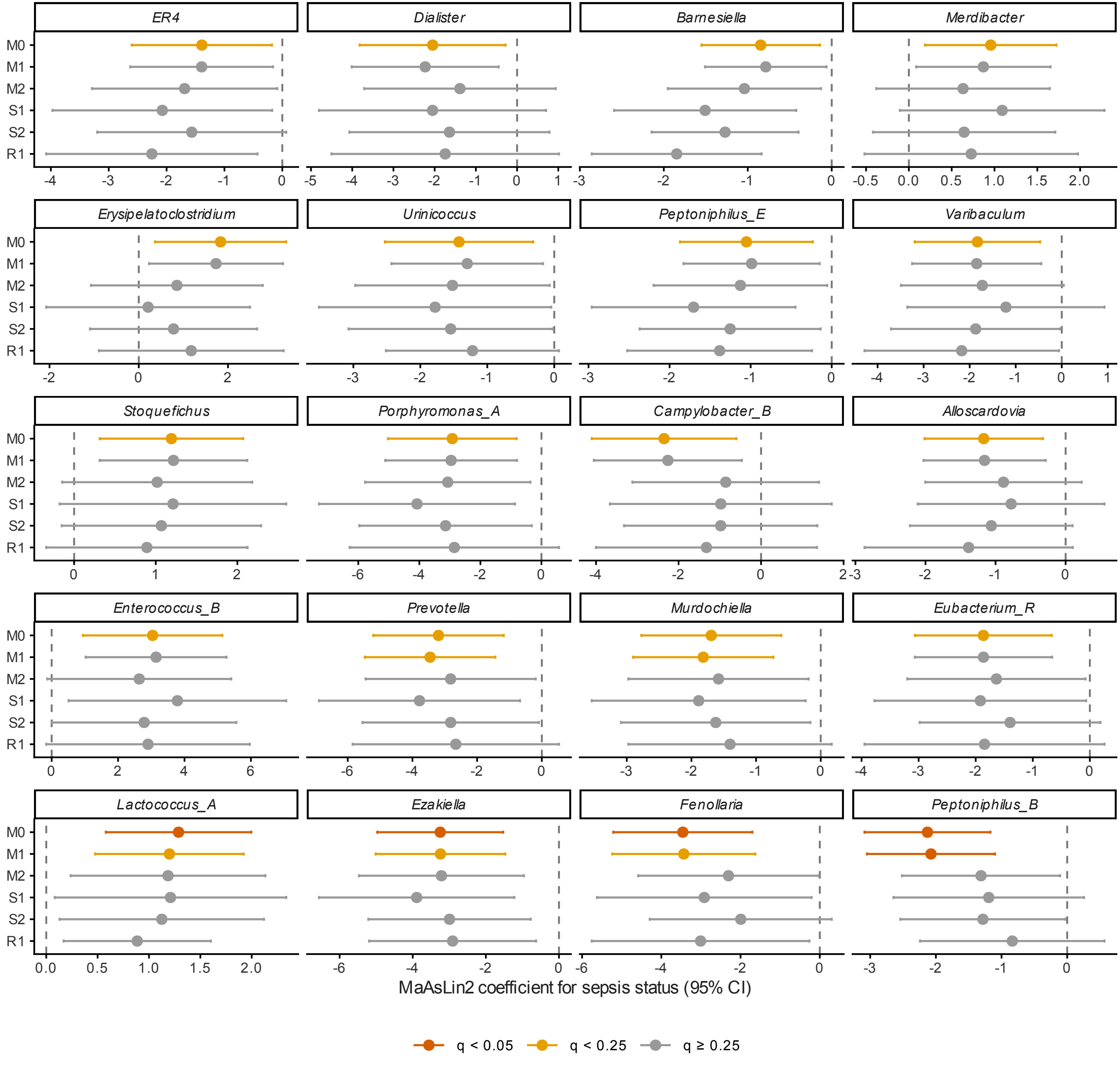
ER4
Dialister
Barnesiella
Merdibacter
Erysipelatoclostridium
Urinicoccus
Peptoniphilus_E
Varibaculum
Stoquefichus
Porphyromonas_A
Campylobacter_B
Alloscardovia
Enterococcus_B
Prevotella
Murdochiella
Eubacterium_R
Lactococcus_A
Ezakiella
Fenollaria
Peptoniphilus_B
M0
M1
M2
S1
S2
R1
MaAsLin2 coefficient for sepsis status (95% CI)
q < 0.05
q < 0.25
q ≥ 0.25
