# Supplementary material for: Gut microbiome diversity, genus-level variation, and associations with clinical features in critically ill patients
Source: Front Med (Lausanne). 2026 Sep 23;13:1903736. doi: 10.3389/fmed.2026.1903736 (PMC13642589; doi:10.3389/fmed.2026.1903736)

Mortality associations across prespecified models

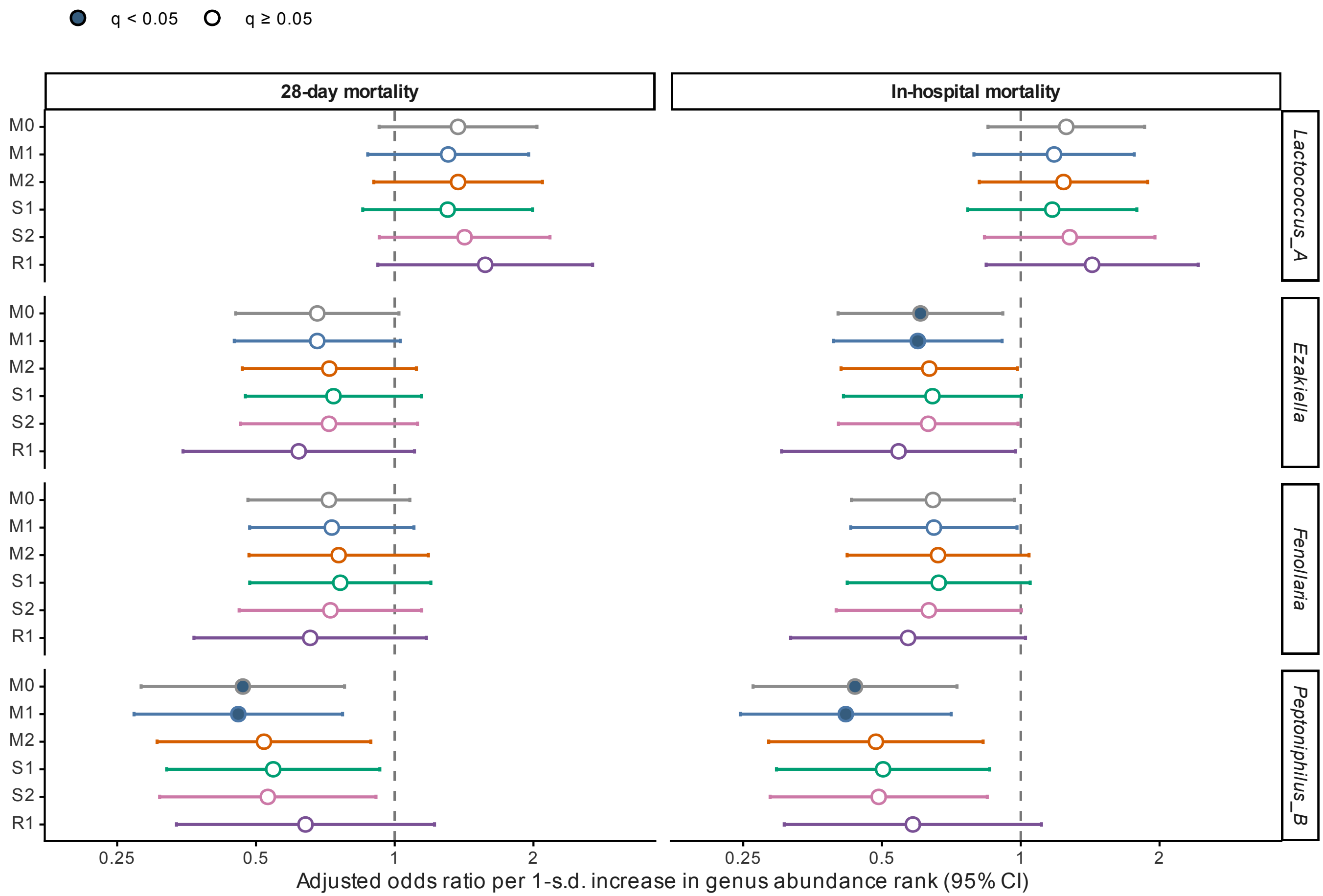

Supplement: Supplementary file 10 [file Image9.pdf]
